# Bacterial cellulose doped with ZnO as a multifunctional bioactive platform for curcumin and propolis immobilization: synthesis, characterization, and wound healing potential

**DOI:** 10.1186/s12934-025-02826-6

**Published:** 2025-08-25

**Authors:** Ghada E. Dawwam, Naglaa Salem El-Sayed, Mona T. Al-Shemy

**Affiliations:** 1 Botany and Microbiology Department, Faculty of Science, Benha University, Benha, 13518 Egypt; 2 Cellulose and Paper Department, National Research Centre, 33 El-Bohouth St. (Former El-Tahrir St.), Dokki, P.O. Box 126220, Giza, Egypt

**Keywords:** Bacterial cellulose (BC), Biomedical applications, *Limosilactobacillus fermentum*, ZnO NPs, Propolis extract (Pp), Curcumin (Cc), Antimicrobial properties

## Abstract

Bacterial cellulose (BC)-based dressings exhibit favorable hydrogel characteristics, including high biocompatibility, moisture regulation, and mechanical adaptability, making them suitable candidates for biomedical applications. In this study, an integrated approach was employed to develop multifunctional, bioactive bionanocomposites. A cellulose-producing bacterial strain, *Limosilactobacillus fermentum* 6BC (accession number OM978241.1), was isolated from spoiled grapes and identified through 16 S rRNA gene sequencing. The structural and chemical characteristics of the synthesized BC were analyzed using Transmission electron microscopy (TEM), scanning electron microscopy (SEM), Fourier-transform infrared spectroscopy (FTIR), and X-ray diffraction (XRD). To enhance the biological functionality of BC, zinc oxide (ZnO) nanoparticles were incorporated via a sono-coprecipitation method to produce ZnO-decorated BC (BCZO). The resulting BCZO was subsequently integrated into a hydroxypropyl ethyl cellulose (HPEC) matrix, forming a bionanoplatform designed for the immobilization of bioactive agents, specifically curcumin (Cc) and propolis extract (Pp). TEM was used to examine the nanoscale distribution of ZnO, while FTIR, XRD, SEM, and EDS were employed to characterize the composite structure and confirm drug incorporation. The fabricated scaffolds were further subjected to comprehensive antimicrobial, antioxidant, cytocompatibility, and in vitro wound healing assessments to evaluate their biological performance. The antimicrobial assays demonstrated effective inhibition of *L. monocytogenes*, *S. aureus*,* E. coli*,* Salmonella* sp., and the fungus *C. albicans*. The bionanoplatforms also exhibited concentration-dependent antioxidant activity in DPPH and ABTS assays (ascorbic acid as control). The cytocompatibility tests on human skin fibroblasts (HFB-4) showed excellent cell viability across all formulations. Among them, Cc/Pp50@BCZO/HPEC displayed the exciting antimicrobial and antioxidant performance, coupled with desirable cytocompatibility. Thus this study offers a systematic framework for engineering cellulose-based bioplatforms as bioactive materials for potential drug delivery and wound-healing applications.

**Supplementary Information:**

The online version contains supplementary material available at 10.1186/s12934-025-02826-6.

## Introduction

Modern wound care has taken a transformative leap forward with the development of moisture-regulating dressings. These innovative materials go beyond the limitations of traditional gauze by actively cultivating a moist environment, an essential condition for efficient and accelerated healing. Through precise moisture management, they encourage key biological processes such as cellular migration, angiogenesis (the formation of new blood vessels), and autolytic debridement. Moreover, these dressings serve a dual purpose as delivery platforms, allowing therapeutic agents like antimicrobials, growth factors, and bioactive healing compounds to be administered directly to the wound site for targeted treatment [1]. These innovations have led to the development of various formulations, including foams [2], nanofibers [3], films [4], sponges [5], and hydrogels [6], each tailored to address specific wound care needs. By maintaining low oxygen levels at the wound site and activating key factors like hypoxia-inducible factor-1, these dressings not only promote healing but also accelerate reepithelialization [7]. Among these, hydrogels have emerged as particularly promising due to their high water content and 3D polymeric network structure, which have proven beneficial in biomedical applications, including wound dressings [8].

The growing prevalence of chronic wounds, exacerbated by an aging population and the increasing threat of antibiotic resistance, underscores the urgency for innovative wound-care solutions [9]. Chronic wounds, often characterized by senescent fibroblast and keratinocyte populations, present significant treatment challenges due to factors like heightened oxidative stress, elevated levels of reactive oxygen species (ROS), and impaired antioxidant pathways [10]. These wounds require advanced dressings that not only promote healing but also effectively combat microbial infections without hindering cellular proliferation [11].

BC, is a natural hydrogel, has gained much attention as a biomaterial for biomedical applications due to its remarkable mechanical properties, nanofiber network structure, biocompatibility, high water retention capacity, and liquid permeability [12]. In addition, BC has high chemical purity compared to plant-derived cellulose, which contains hemicellulose and lignin [13]. These potential characteristics make BC an ideal candidate for oil separation [14], skin wound dressings [15], drug delivery, and tissue engineering [16].

Despite its advantages, BC lacks intrinsic desired biological properties such as antimicrobial, antioxidant, and anti-inflammatory activities [17]. Functionalization of BC or decoration with nanoparticles can tackle these disadvantages. For instance, the incorporation of metal/metal oxide nanoparticles into BC generates wound dressings with improved antimicrobial properties that can be effective in eradicating different microbial strains, biofilm formation, and combating microbial resistance to antibiotics [18]. These composites exhibit improved moisture retention, gas permeability, and drug delivery capabilities, making them ideal candidates for next-generation wound dressings [19]. On the other hand, commercial translation remains limited due to scalability challenges and regulatory hurdles, necessitating further research to optimize production and functionality [20].

Zinc oxide nanoparticles (ZnO NPs) are important metal oxide nanoparticles and are intensively studied for their photooxidative characteristics that are widely employed in many industrial fields [21, 22]. When ZnO NPs are used in the manufacturing of rubber, they improve its resistance to corrosion, aging, and UV light, supporting its performance [23]. Also, ZnO was used in personal care products, such as cosmetics and sunscreen, due to its UV-shielding ability [24]. Thus, the prospective microbicidal properties of ZnO and excellent UV absorption ability enabled its utilization in various biomedical applications, such as antimicrobial, anticancer, antineoplastic, wound healing, angiogenic, and antioxidant properties.

Several research papers have reported the functionalization of BC with ZnO NPs and evaluated its performance either alone or with other active gradients such as drugs, and biopolymers. Khalid et al. integrated ZnO NPs into BC hydrogel to develop BC-zinc oxide (BC-ZnO) nanocomposites that were employed as dressings for burn healing. The nanocomposites successfully inhibited the growth of pathogens that may infect the burns. Moreover, they showed remarkable healing and supported the regeneration of injured tissue in mice models [25].

In a different approach, Kai et al. fabricated biocompatible composite hydrogels composed of ZnO NPs infused into hydrogels made of chitosan cross-linked oxidized BC (ZnO@BCCS). the mechanical testing for the nanocomposites indicated its 15 times improvement in the compressive stress at 60% deformation than the hydrogel without ZnO NPs, and a maximum tensile stress is 880 kPa. 0.05M ZnO@BCCS hydrogels exerted a maximum inhibition rate of 78.9% and 77.6% against *Escherichia coli* and *Staphylococcus aureus*, respectively. The prepared ZnO@BCCS composites had outstanding biocompatibility and supported the MC3T3-E1 cell proliferation [26].

In a recent study, Saleh et al. successfully produced BC from *Lactiplantibacillus plantarum* AS.6 under static conditions, and the resulting BC hydrogel was further decorated with zinc oxide ZnO-NPs prepared via ex-situ ultrasonication. The sheets displayed antimicrobial potential with inhibition zones of 21, 18, 19, and 14 mm for *S. typhimurium*, *E. coli*, *S. mutans*, and *C. albicans*, respectively [27]. Also, Mocanu et al. immobilized the ethanolic extract of propolis (Pp) (*Dolj County*, *Romania* ) into ZnO-modified BC films to prepare biodegradable food packaging material with antimicrobial and antioxidant properties [28].

Curcumin (Cc) exhibits remarkably potent anti-inflammatory effects that rival those of both steroidal and nonsteroidal drugs such as indomethacin and phenylbutazone, yet without their dangerous side effects [29]. Its action is mediated primarily by inhibiting the induction of key inflammatory enzymes like cyclooxygenase-2 (COX-2), lipoxygenase (LOX), and inducible nitric oxide synthase (iNOS). Moreover, Cc suppresses the production of pro-inflammatory cytokines, including interferon-γ and tumor necrosis factor, and modulates the activity of crucial transcription factors, notably NF-κB and AP-1 [30].

Cc loading into BC has been explored as an emerging packaging and wound dressing material. The immersion of BC films into 0.5 and 1.0 mg/mL of Cc solutions augmented their antifungal activities against *Aspergillus niger.* in addition, Cc-loaded BC films revealed selective antiproliferation ability against malignant melanoma (A375 cells) without significant toxicity against normal human keratinocytes and human dermal fibroblasts [31]. In addition, Dias et al. [32] showed that silver selenium nanoparticles complexed with Cc (Cur-AgSeNPs) were immobilized into BC at different molar concentrations. The assembled composite films revealed efficacy as antimicrobial antioxidant dressings (scavenging activity of 63.92 ± 0.76%). Theacceptable hemolysis rate (10%) and cytocompatibility towards L929 fibroblast cells suggest its possible use in skin healing fabrics.

Recent studies have explored the synergistic effects of Cc and zinc oxide (ZnO) nanoparticles in wound healing applications. For instance, Bhutta et al. [33] demonstrated that a nano ZnO/Cc composite significantly accelerated healing in third-degree burns by enhancing collagen deposition, angiogenesis, and epithelialization compared to individual components. Similarly, Kalirajan and Palanisamy [34] embedded ZnO–Cc nanocomposites into collagen scaffolds, achieving scarless skin regeneration through upregulation of TGF-β3 and vascularization. While these findings underscore the therapeutic potential of Cc-ZnO systems, most studies focus on isolated formulations or animal models, with limited integration into bacterial cellulose-based platforms. This gap highlights the need for advanced biomaterials, such as bacterial cellulose nanocomposites, that combine structural support with bioactive functionality for enhanced wound management.

In this study, we report for the first time the biosynthesis of bacterial cellulose from spoiled fruit biomass by *Limosilactobacillus fermentum* 6BC and its in situ decoration with ZnO NPs. The ZnO NPs were prepared onto the BC via a newly developed sono-coprecipitation method, which, to the best of our knowledge, is the first application of this approach. This technique yielded uniformly sized, high-purity ZnO NPs ready for subsequent incorporation into the bacterial cellulose matrix. We then evaluated how integration of ZnO, Cc, Pp extract, and a Cc/Pp mixture into the BC matrix enhances antioxidant activity, antimicrobial potency, and wound-healing performance. We hypothesize that these novel nanocomposites will outperform conventional BC systems, offering a multifunctional platform for advanced wound dressings. Thus, this study aimed to develop a cost-effective, multifunctional bionanoplatform by integrating ZnO nanoparticles, Cc, and Pp into a bacterial cellulose (BC) matrix synthesized from.

## Experimental

### Materials

Pp was purchased from Imtenan, Egypt; Cc was purchased from Santa Cruz, San Diego, California, USA; zinc acetate from Laboratory Rasayan; ethanol, ethyl acetate, NaOH, tris-HCl buffer, and hydroxypropyl ethyl cellulose (HPEC) were purchased from Sigma Aldrich. Fruits were collected from local markets in El-Qalubia district, Egypt. Nutrient broth media and Mueller-Hinton Agar media were purchased from Oxoid Ltd., England. ABTS (2,2’-azino-bis (3-ethylbenzothiazoline-6-sulfonic acid) and DPPH (2,2-diphenyl-1-picrylhydrazyl) were purchased from Sigma Aldrich. For the cytocompatibility assay, the normal human fibroblasts cell line HFB4 was purchased from VACSERA, Egypt.

### Methods

#### **Production of BC**

##### Isolation of cellulose-producing bacteria

Each fruit sample (apple, banana, guava, grapes, mango, orange, pomegranate, and sweet lime) measured by weight (7 g) and added to a 250 mL Erlenmeyer flask along with 50 mL of Hestrin-Schramm (HS) broth media (D-glucose 20 g/L, yeast extract 5 g/L, peptone 5 g/L, disodium phosphate 2.7 g/L, and citric acid 1.15 g/L), as a static incubator. The incubation period was 10 days at 30 °C.

##### Screening of cellulose producers

At the air-liquid interface, pellicle production was detected in all flasks. The flasks that developed pellicle growth were chosen, and the culture was isolated by streaking it on HS agar plates many times.

##### Detection of cellulose production and quantification

The formed pellicle was collected and washed three times with water. After that, the pellicle was cured with 4 N NaOH for 24 h at room temperature, followed by treatment with a 6% acetic acid solution to neutralize the NaOH. Three or four further washes with water were performed. To find the BC production, the purified pellicle was allowed to dry at room temperature until it reached a consistent weight. BC yield (g/L) was measured according to the method described by Farag et al. [35].

##### Molecular identification (DNA extraction and analysis of molecular phylogeny using the 16 S rRNA gene sequence)

The genomic DNA for the isolate code 6BC was obtained following the instructions provided by the GspinTM Total Extraction kits. Bacterial DNA was extracted from cells cultured in Luria–Bertani (LB) broth. A thermocycler (Biometra, Germany) was employed to amplify the 16S rRNA genes of the new isolates using the universal primers 27F 5’ (AGA GTT TGA TCM TGG CTC AG) 3’ and 1492R 5’ (TAC GGY TAC CTT GTT ACG ACT T) 3’, as specified by Pandey et al. [36]. The PCR amplification protocol consists of an initial denaturation at 95 °C for 5 min, followed by 30 cycles comprising denaturation at 95 °C for 30 s, annealing at 55 °C for 2 min, and extension at 68 °C for 1.5 min, concluding with a final extension at 68 °C for 10 min. The Montage PCR clean-up kit (Millipore) was utilized to purify PCR products. The Big Dye Terminator Cycle Sequencing Kit v.3.1 (Applied Biosystems, USA) was utilized, and the sequencing products were analyzed using an Applied Biosystems model 3730XL automated DNA sequencing system (Applied Biosystems, USA) at Macrogen, Inc., Seoul, Korea. The Sequence Similarity Search was conducted for the 16 S rDNA sequence utilizing an online search tool (http://www.ncbi.nlm.nih.gov/blast/). The unidentified organism was characterized via maximum aligned sequence analysis using a BLAST search and subsequently deposited in GenBank with accession number OM978241.1.

#### Decoration of BC with ZnO NPs (BCZO) via sono-coprecipitation synthesis

The sono-coprecipitation method, based on the procedure reported by Dawwam et al. [37] with significant modifications, was employed for the in situ synthesis of ZnO nanostructures within BC. Initially, 2.5 g of BC was dispersed in 200 mL of distilled water and homogenized for 15 min. Subsequently, 0.125 g of NaOH was added to the suspension, which was then heated at 50 °C for 2 h. The resulting mixture was dialyzed against distilled water until the excess alkali was completely removed, yielding a final pH of approximately 7.5.

Next, 0.5 g of a Zn(CH_2_COO)_2_ solution was introduced into the pre-existing suspension. The mixture was then subjected to ultrasonication using a probe sonicator (60 W, ten pulses per second at 220 V) for 30 min. The synthesized BCZO nanocomposite was dialyzed against deionized water until the suspension’s conductivity dropped below 4 µS/cm, approaching the conductivity of pure water (∼ 0.038 µS/cm), indicating effective removal of residual ions and unreacted precursors [38]. Finally, the nanocomposite hydrogel was collected, freeze-dried, and the resulting powder was stored at 4 °C for future applications.

#### Extraction of Pp

20 g of dry Pp was placed in a 500 mL round flask containing 200 mL of absolute ethanol and stirred for 24 h at room temperature using a magnetic stirrer. Then, the filtrate was collected by centrifugation. The resulting residue was collected and resuspended in 200 mL of ethanol and left for an additional 24 h while stirring. The suspension was further centrifuged, the filtrate was collected and mixed with the ethanolic fraction, and the solvents were evaporated using rotavapor. The resulting viscous paste was collected, freeze-dried, and stored at 4 °C for further use.

#### Preparation of BCZO/HPEC bionanoplatform

A 10% HPEC solution was prepared by dissolving 10 g of HPEC in 100 mL of distilled H_2_O. Subsequently, 100 mg of BCZO was suspended in 10 mL of distilled H_2_O and added to 9 mL of the HPEC solution (900 mg). The mixture underwent probe sonication (60 W, ten pulses per second at 220 V) for 10 min in an ice-water bath to ensure the complete dispersion of ZnO@BC within the HPEC hydrogel. Finally, the hydrogel was freeze-dried for 72 h for further characterization.

#### Immobilization of Cc and Pp into BCZO/HPEC Bionanoplatform

The proper amounts of Cc and Pp (Table 1) were dissolved in 5 mL of ethanol and added dropwise to freshly prepared BCZO/HPEC hydrogel. The blend was homogenously mixed using a probe sonicator (60 W, 10/sec pulse at 220 V) for 10 min in an ice-water bath, followed by magnetic stirring at 500 rpm for an additional 20 min. Finally, the different nanoplatforms were freeze-dried and stored at 4 °C for further use.

The drug entrapment efficiency for BCZO, Cc, and Pp by HPEC was calculated according to the method described by Judefeind and de Villiers [39].

1 $$ {\text{Drug loading }}\left( \% \right) = \left( {W_{d} - W_{0} } \right)/W_{0} \cdot 100 $$

where W_d_ is the weight of the drug-loaded sample, W_o_ is the weight of the original (drug-free) sample.

**Table 1:** Composition of different BC-derived bionanoplatforms with drug loading and encapsulation efficiency

Bionanoplatform	BCZO (mg)	HPEC (mg)	Pp (mg)	Cc (mg)	Drug loading (%)	Encapsulation efficiency (%)
BCZO	100	0	0	0	0.0	–
BCZO/HPEC	100	900	0	0	0.0	–
Cc@BCZO/HPEC	100	900	0	100	10.0	100
Pp@BCZO/HPEC	100	900	100	0	10.0	100
Cc/Pp50@BCZO/HPEC	100	900	50	50	10.0	100
Cc/Pp100@BCZO/HPEC	100	900	100	100	20.0	100

### Characterizations

The structural and surface characterizations of synthesized neat BC and its analogue-derived bionanoplatforms (e.g., BCZO, BCZO/HPEC, Cc@BCZO/HPEC, Pp@BCZO/HPEC, Cc/Pp50@BCZO/HPEC, and Cc/Pp100@BCZO/HPEC) were conducted using various analytical techniques:

- Fourier Transform Infrared (FT-IR) spectroscopy was performed using a Nicolet Impact-400 FT-IR spectrophotometer within the spectral range of 400–4000 cm^−1^.
- The XRD patterns of the as-prepared lyophilized materials were analyzed using a Malvern Panalytical Empyrean X-ray diffractometer (the Netherlands), with an angle of incident monochromatic X-ray in the range of 2θ = 5°–80°.
  The crystallinity index was calculated based on the following formula [40]:
  2 $$ CrI(\% ) = A_{c} /\left( {A_{c} + A_{{am}} } \right) \cdot 100 $$
- where A_c_ is the area under the crystalline peaks and A_am_ is the remaining (amorphous) area.
- SEM micrographs were obtained using a Quanta FEG-250 high-resolution scanning electron microscope (Waltham, MA, USA) operating at an accelerating voltage of 20 kV. Before imaging, samples were sputter-coated with a thin layer of gold to improve surface conductivity and contrast. The same instrument was utilized for elemental analysis via energy-dispersive X-ray spectroscopy (EDS) to characterize the fabricated materials. Images were recorded at magnifications of 200×, 1,000×, 3,000×, 5,000×, 6,000×, and 12,000×, 24,000×, enabling visualization of both macro and microstructural features, including surface morphology, dispersion and distribution of embedded nanocomposites, porosity, and fibre interactions. Under these imaging conditions, the estimated spatial resolution ranged from ~ 5 nm to ~ 1–2 nm, depending on magnification and working distance. Moreover, transmission electron microscopy (TEM) micrographs were captured with high-resolution JEOL JEM-2100 (Japan). The as-prepared suspensions were diluted 10 times before being dried on a microgrid covered with a thin carbon film (≈ 200 nm).

### Biological evaluations

#### Antimicrobial activity

BCZO, BCZO/HPEC, Cc@BCZO/HPEC, Pp@BCZO/HPEC, Cc/Pp50@BCZO/HPEC, Cc/Pp100@BCZO/HPEC, Cc, and Pp were tested against Gram-positive bacteria (*L. monocytogenes* ATCC 19155, *Staphylococcus aureus* ATCC 43300), Gram-negative bacteria (*E.coli* ATCC 8739, *Salmonella* sp. ATCC 14028), and unicellular fungi (*Candida albicans* ATCC 10231); via the agar diffusion approach according to Dawwam et al. [37]. The bacteria were cultured for 24 h at 37 °C in a liquid nutrient medium using a shaker bed set at 200 rpm. The bacteria (1.5 × 10^8^ CFU) were swabbed on Mueller-Hinton agar plates. Wells (with a diameter of 7 mm) cut into the agar plates were used. 200 µL of 20 mg/mL of tested samples dissolved in DMSO (10%) were added to the wells. DMSO (10%) was used as a negative control, whereas curcumin and propolis were used as positive controls. The agar plates were kept at 37 °C for 24 h. Triplicate experiments were performed for each strain examined, and the millimeter-sized inhibitory zones surrounding the discs were measured.

#### Antioxidant activity assay

##### DPPH assay

The DPPH scavenging ability of BCZO, BCZO/HPEC, Cc@BCZO/HPEC, Pp@BCZO/HPEC, Cc/Pp50@BCZO/HPEC, and Cc/Pp100@BCZO/HPEC at concentrations of (10, 20, 40, 60, 80, and 100 µg/mL), as compared with control (L. ascorbic acid), was measured using the DPPH method described by Khalil et al. [41]. In brief, 0.1 mL of the test sample was mixed with 1 mL of a methanolic solution of 0.06 mM DPPH and incubated in the dark for 30 min. The absorbance was measured at 517 nm, and the percentage of DPPH scavenging was calculated according to Eq. 3 as follows:

3 $$ \begin{gathered} {\text{DPPH scavenging percentage }}(\% ) = \hfill \\ \left[ {\left( {A_{{control}} - A_{{testsample}} } \right)/A_{{control}} } \right] \cdot 100 \hfill \\ \end{gathered} $$

where A_control_ refers to the absorbance of the control, A_test sample_ refers to the absorbance of the test sample.

##### ABTS scavenging assay

The ABTS free radical scavenging assay was also employed to determine the antioxidant activity for the different platforms at concentrations of (10, 20, 40, 60, 80, and 100 µg/mL), following the protocol described by Pellegrini et al. [42]. Briefly, 0.1 mL of the test sample was added to 1 mL of activated ABTS solution (7 mM) and incubated for 7–10 min. After incubation, the absorbance was determined at 734 nm, and the percentage of ABTS radical inhibition was calculated using Eq. 4.

4 $$\begin{gathered} {\text{ABTS free radical scavenging percentage }}(\%) \hfill \\ = \left[ {\left( {A_{{control}} - A_{{testsample}} } \right)/A_{{control}} } \right] \cdot 100 \hfill \\ \end{gathered} $$

where A_control_ refers to the absorbance of the control, A_test sample_ refers to the absorbance of the test sample.

#### In vitro cytocompatibility assay

Cultures of normal human skin cells (HFB-4) were maintained in a humidified cell incubator at 37 °C with 5% CO_2_ and 95% air. The medium used for the cells was RPMI with 1% penicillin-streptomycin, 1% non-essential amino acids, and 10% fetal bovine serum (FBS). After reaching approximately 70% confluence in 96-well plates, the cells were cultured for 24 h following treatment with serial concentrations of the tested samples: 1000, 500, 250, 125, 62.5, and 31.25 µg/mL. DMSO was used as a control. The plates were put in a humidified CO_2_ incubator and incubated at 37 °C for 4 h after the medium was withdrawn and replaced with 120 µL and 30 µL of MTT 3-(4,5-dimethylthiazol-2yl)-2,5-diphenyl tetrazolium bromide solution, respectively, at a concentration of 5 mg/ml. To facilitate color development, 100 µL of DMSO was added to each well after removing the supernatant. Subsequently, the plates were incubated under shaking conditions for 10 min. Lastly, a microplate reader detected the absorbance at 540 nm [43].

The most straightforward technique to determine the IC_50_ is to plot the data x-y and fit it with a straight line (linear regression). To determine the amount of viable cells and their percentage of viability, the following formulas were utilized (Eqs. 5 and 6):

5 $$ Cell viability (\% ) = \left( {Test OD} \right)/\left( {Control OD} \right) \times 100 $$

6 $$ Inhibition \% = 100 - Viability\% 100 $$

#### In vitro wound healing assay

**Scheme 1:**
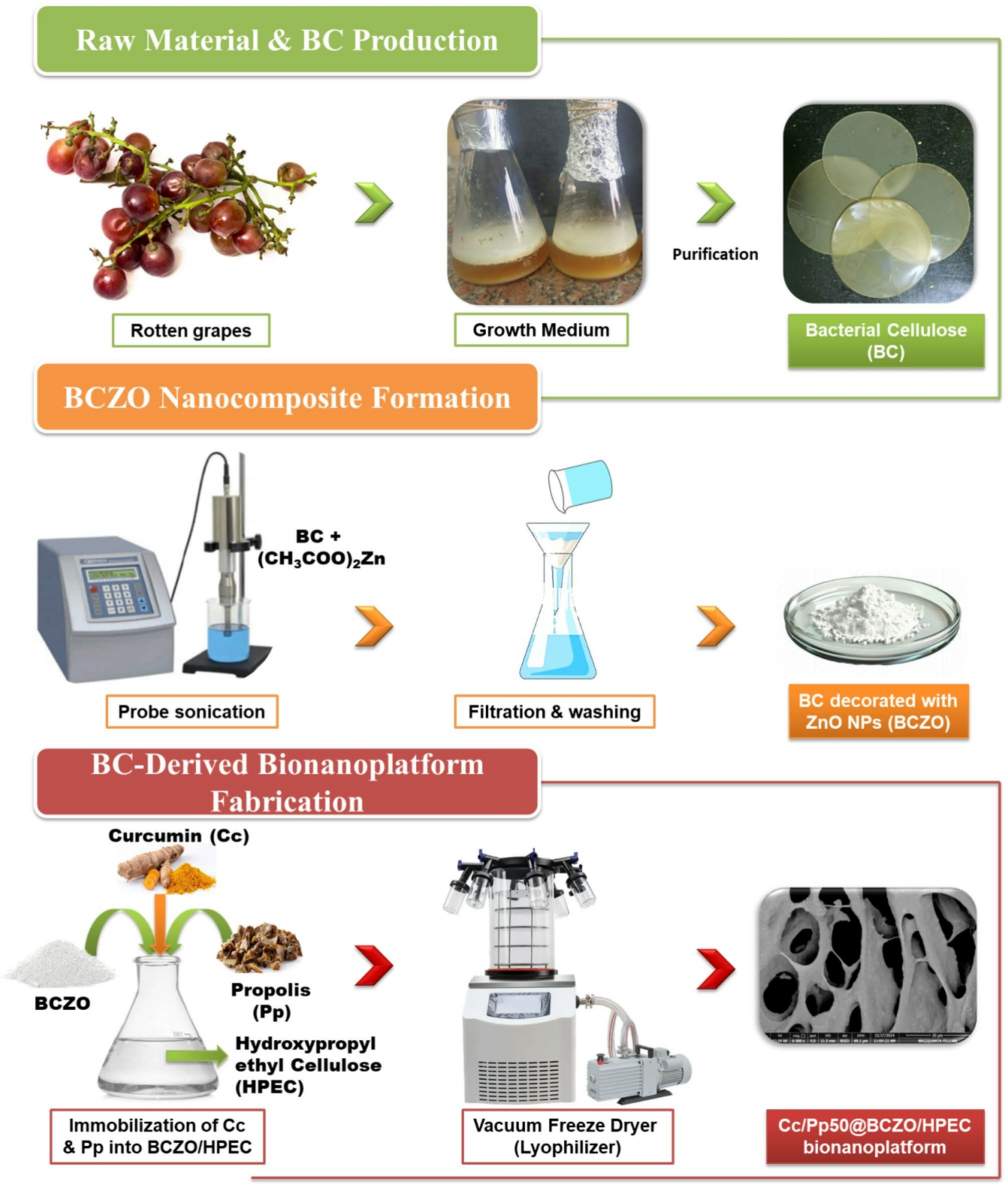
The main fabrication steps involved in developing BC, BCZO nanocomposite, and Cc/Pp50@BCZO/HPEC bionanoplatform

A culture of normal human skin cells was established on 24-well plates. The cells were then placed in a humidified cell incubator with 5% CO_2_ and 95% air at 37 °C. The medium used for the culture was RPMI supplemented with 1% penicillin-streptomycin, 1% nonessential amino acids, and 10% fetal bovine serum (FBS). Two washes with PBS were performed on the cell monolayers after incubation. A pipette tip containing 10–100 µL was used to make a vertical scratch in every well. After removing the detached cells by rinsing them in PBS, each well was filled with low-serum fresh media, and an image of the scratch was obtained at zero time. Following 30 min of UV sterilization, the Cc/Pp50@BCZO/HPEC sample (20 mg) was utilized. A narrow linear scratch was carefully introduced into a confluent monolayer of cells following the method described by El-Sayed et al. [44]. Using a ZOE Fluorescent Cell Imager fluorescent microscope (BIO-RAD, USA), images of the scratch closure were captured after 48 h. Using the open-source Image J program, we can determine the scratch width [45]. The main fabrication steps and analysis for the material under investigation are shown in Scheme 1.

### Statistical analysis

Data were statistically determined using the IBM^®^ SPSS^®^ Statistics software version 21 on the premise of Duncan’s multiple range test at the 5% level. All analyses were performed in triplicate.

## Results and discussion

### Production of BC

#### Isolation and identification of BC producing bacteria

Isolating bacteria that produce BC often involves using spoiled fruits. A gelatinous mat-like structure (BC pellicle) formed at the air-liquid interface was detected in the flask with spoiled grapes, which was reported as a favorable outcome for BC production. Isolates were evaluated for BC production using HS medium after being purified by serial dilution to yield 6 samples coded (BC1, BC2, BC3, BC4, BC5, and BC6) with production of (1.3, 0.9, 1.7, 0.7, 1.9, and 2.8 g/L), respectively, of pellicle weight. Isolate code BC6 was chosen for further studies.

#### Molecular identification

**Fig. 1:**
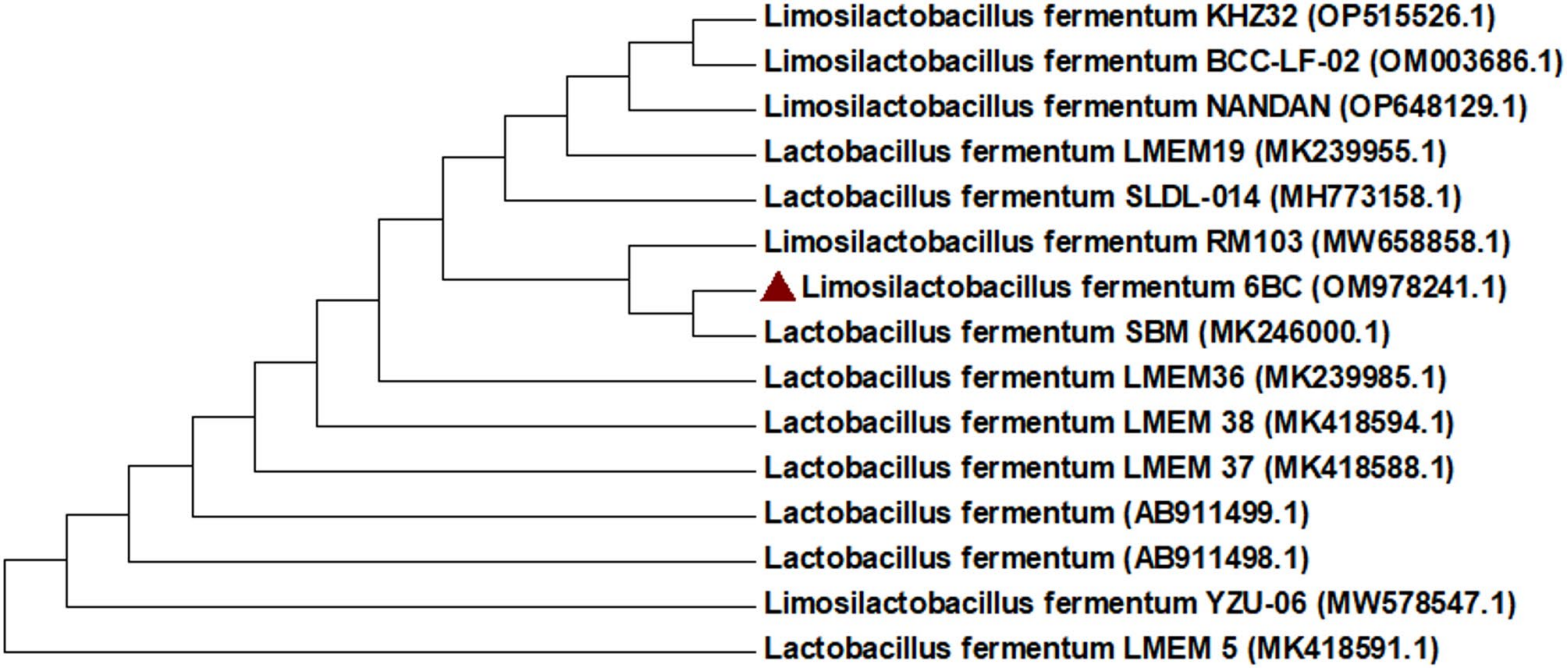
A Phylogenetic tree was constructed using the neighbor-joining method. The bootstrap consensus tree inferred from 1,000 replicates is used to represent the evolutionary history of the analyzed taxa. The final dataset comprised a total of 681 positions. Evolutionary analyses were conducted in MEGA7

A phylogenetic tree between the chosen strains and other strains is presented in Fig. 1. The most promising isolate 6BC was identified by molecular identification using 16 S rRNA as *Limosilactobacillus fermentum* 6BC with 100% similarity and recorded in GenBank under accession numbers (OM978241.1). In this regard, Saleh et al. [46] isolated an effective BC-producing strain from rotten apples and identified it as the Gram-positive *Lactiplantibacillus plantarum (L. plantarum* AS.6) strain.

#### Depictions of produced BC

**Fig. 2:**
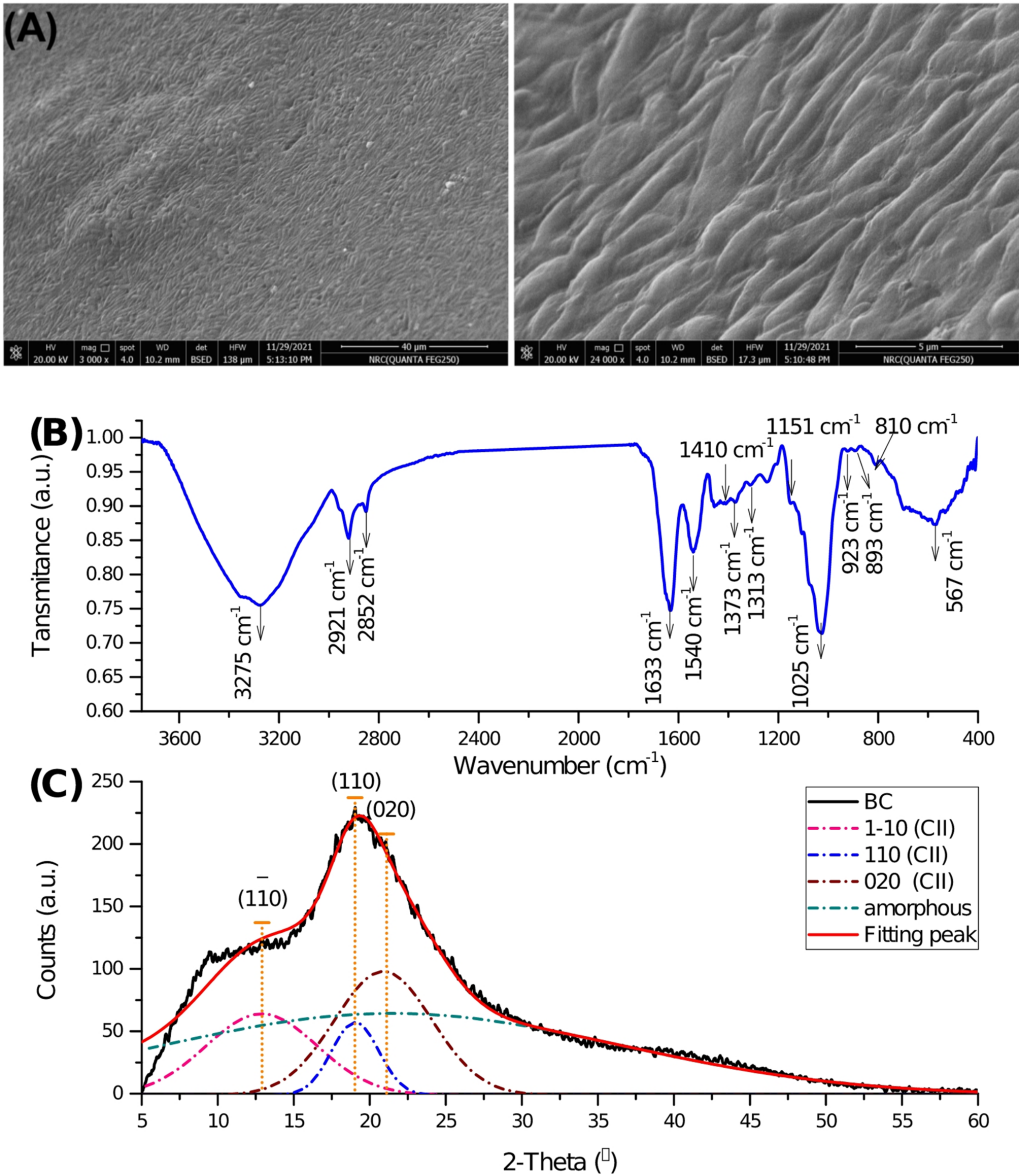
Characterization of BC: (A) SEM micrographs with magnifications of 3000× and 24,000×, respectively; (B) FTIR spectrum revealing functional groups typical of cellulose; (C) XRD pattern with deconvoluted peaks indicating Cellulose II crystalline structure

The SEM image (A) reveals two micrographs of BC at two different magnifications, at 3000X and 240,000X. The surface seems smooth, with a subtle rippling texture in the lower magnification photograph. The higher-magnification picture shows an intricate web of ribbon-like fibers, strings of tightly packed fibers that are somewhat wavy or undulating (Fig. 2A).

Figure 2B presents the FTIR spectrum of BC, revealing patterns analogous to those observed for BC, the sample’s primary component. The initial absorption peak at 3277 cm^− 1^ can be attributed to hydroxyl groups (-OH) stretching vibrations resulting from both carbohydrates and absorbed water [17]. The absorption peaks in the 2990–2800 cm^− 1^ region are related to the symmetric and asymmetric stretching modes of C–H in the methylene (CH_2_) and methyl (CH_3_) functional groups linked to cellulose [47, 48]. The 1632 cm^− 1^ peak is attributed to the bending vibrations of O-H bonds, likely associated with absorbed moisture in the sample [49]. Meanwhile, the 1540 cm^− 1^ peak is linked to C-H bending vibrations [50, 51]. In BC, absorption peaks at 1410, 1373, and 1313 cm^− 1^ indicate various vibrational modes within the polysaccharide structure. H-C-H and O-C-H bending vibrations at 1410 cm^− 1^ indicate organic functional groups, while C-H and COH bending at 1373 cm^− 1^ indicate cellulose backbone structural integrity. The 1313 cm^− 1^ signal is associated with CH_2_ wagging and COH bending of cellulose, which are found in BC’s polysaccharide framework [51]. These absorption bands reveal BC composition and interactions, especially when modified or exposed to environmental factors [52, 53]. The 1200–840 cm^− 1^ band in the BC spectrum has a significant absorption peak, mainly ascribed to the stretching vibrations of carbohydrate polymers and their functional groups. These encompass C–H stretching, C–OH stretching, and C–O–C stretching. These peaks are essential in defining the structural composition of BC and its interactions with adjacent molecules. Peaks in this region were accurately ascribed to glucose vibrational modes originating from the cellulose structure in BC [54, 55]. The finding also indicated that the sugar units’ α- and β-configurations might be responsible for the weak absorption bands at 893 cm^− 1^ and 923 cm^− 1^, respectively [56].

The XRD diffraction pattern presented in Fig. 2C reveals that BC exhibited two overlapping intense peaks at 2θ = 19.25 (110) and 2θ = 21.54 (020), in addition to a smaller peak at 2θ = 12.93 (1̅1̅0), which aligns with the CII allomorph structure [58]. This proves that the commonly observed BC CI allomorph was converted into the CII by mercerizing BC during the purification process that involved soaking the BC pellicle in 4 N NaOH at room temperature for 24 h. Many studies have examined the process of mercerization, whereas Na-CI or Na-CII can be generated depending on the alkali content, temperature, and subsequent treatments [59, 60]. The mercerization mechanism can be summarized as follows: at room temperature, expanding microfibrils and breaking certain hydrogen bonds to replace them with sodium atoms are the outcomes of penetrating native crystals with a concentrated NaOH solution. Accordingly, a Na-cellulose complex is generated, and the cellulose crystallite is enlarged. Henceforth, the lateral space between parallel cellulose strands increases, and a coiled conformation is formed when enlarged microfibrils relax and arrange themselves. This new cellulose polymorph molecule allows for stacking hydrophobic planes by making available chains of opposing polarity, which arrange themselves antiparallel [58,61]. The BC exhibits a *CrI* of 42.93%, with crystallite sizes (*D*) in the directions perpendicular to the planes 1̅1̅0, 110, and 020 measuring 0.93 nm, 2.20 nm, and 1.07 nm, respectively. The corresponding lattice spacings (d-spacings) are 6.86 Å, 4.65 Å, and 4.25 Å. The diffraction data obtained are comparable to those reported by Chiriac et al. in their alkali-treated dissolving pulp study [62].

**Fig. 3:**
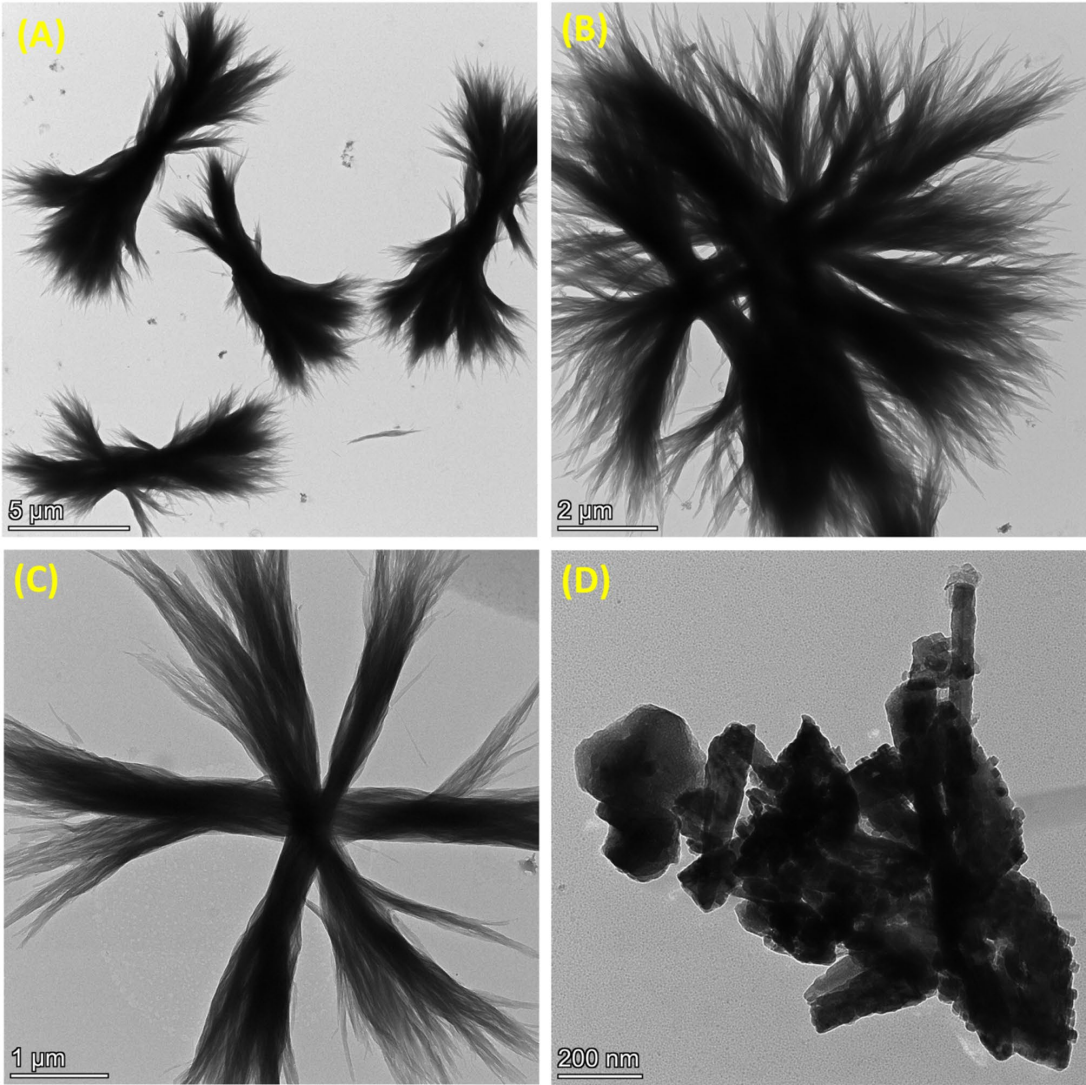
TEM Micrographs of BC and BCZO Nanocomposite: (A, B) BC imaged with scale bars of 5 μm and 2 μm, respectively. (C, D) BCZO nanocomposite visualized at 1 μm and 200 nm scale bars, respectively

Figure 3 presents the TEM micrographs of BC and the BC-ZnO (BCZO) nanocomposite, providing detailed insights into their morphologies. Images (A) and (B) show the native BC structure at scale bars of 5 μm and 2 μm, respectively. These images reveal a dense mass of fine, dendritic fibers arranged in a bush-like architecture, with an average width of 26.67 ± 6.88 nm and lengths ranging from 29 to 48 μm. The three-dimensional network consists of numerous thin nanofibers that radiate outward from central nodes or follow a branched axis, forming a highly entangled and porous matrix.

Overall, BC exhibits a complex, delicate, and highly branched nanofibrillar architecture. Its structure is composed of an interconnected web of ultrafine cellulose fibers, typically 20–100 nm in diameter. This nanofibrous network offers a high surface area, excellent water-holding capacity, and notable mechanical strength. The extensive branching and entanglement of fibers enhance the material’s flexibility, crystallinity, and biocompatibility properties that make BC an ideal candidate for nanocomposite development, biomedical scaffolds, and various advanced material applications.

### Depiction of fabricated BCZO and BC-derived Bionanoplatform

The TEM analysis of the BCZO nanocomposite synthesized via the sono-coprecipitation method reveals distinct morphological and structural transformations compared to pristine BC. While neat BC exhibited irregular and randomly oriented nano fibrillar networks, BCZO displays a more organized and symmetrical star-like morphology (Fig. 3C), suggesting that the incorporation of ZnO nanoparticles influences the self-assembly or nucleation behavior of the cellulose scaffold.

At higher magnifications (Fig. 3D), ZnO nanoparticle aggregates become distinguishable, with some particles adopting a square-like geometry and sizes ranging from 1.21 to 13.50 nm. This variability in particle morphology and size is typical of ZnO synthesized via sonochemical routes, where rapid nucleation under localized high-energy conditions can produce a range of nanostructures. The square-shaped nanoparticles observed suggest well-defined crystalline growth, possibly indicative of controlled crystallization in the presence of the BC matrix, which may act as both a template and stabilizer.

The incorporation of BCZO into hydroxypropyl ethyl cellulose (HPEC) at a fixed 10% weight ratio leads to the formation of a bionanoplatform (BCZO/HPEC) with enhanced functional potential. HPEC, a cellulose ether derivative, not only offers excellent film-forming and emulsifying properties but also promotes biocompatibility and colloidal stability, critical parameters for biomedical or food-related applications. Its ability to integrate with nanostructures enables uniform dispersion and prevents nanoparticle aggregation, thereby enhancing the overall homogeneity and mechanical integrity of the composite.

**Scheme 2:**
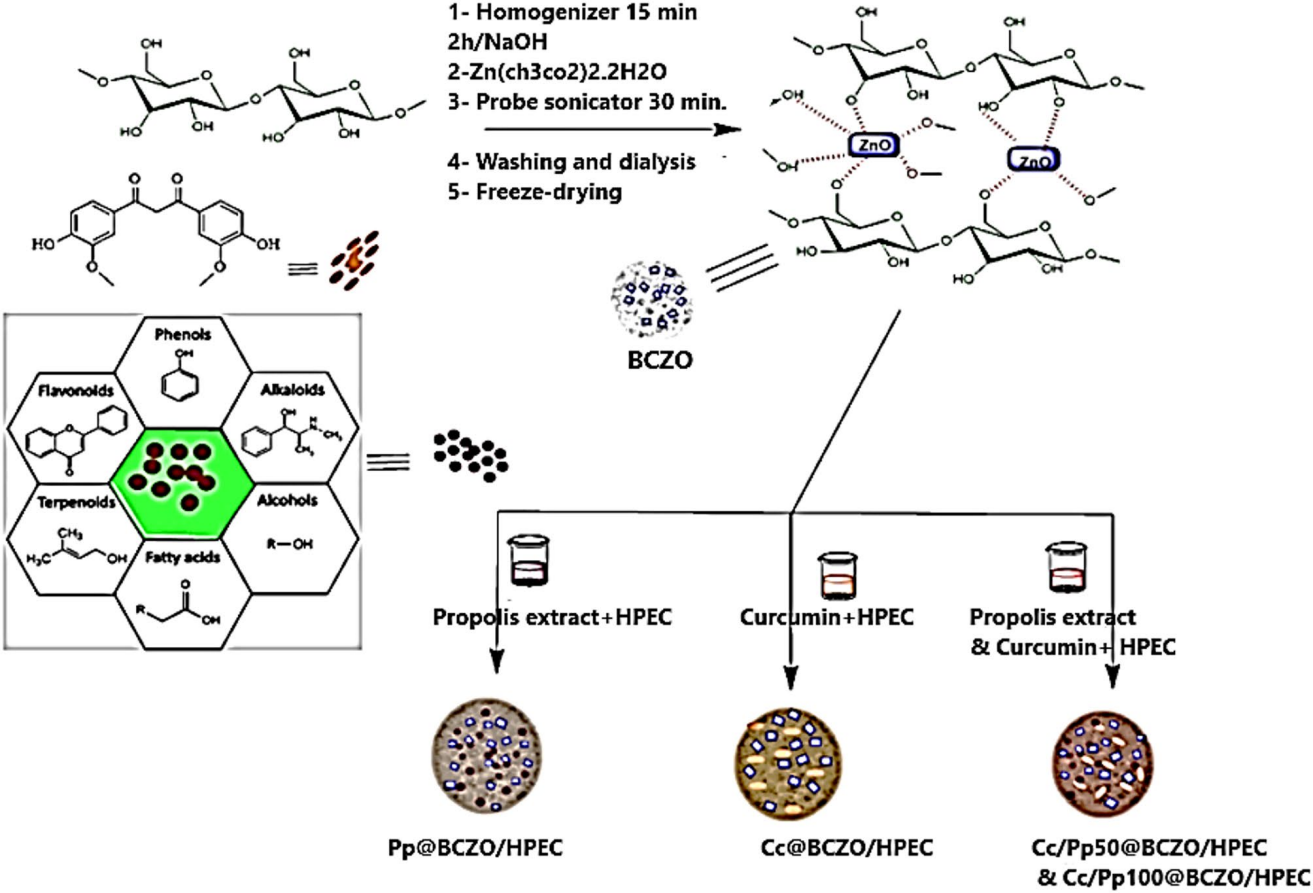
Integration of Pp and Cc into a BCZO bioactive system for enhanced wound healing efficacy

The synergistic effect of combining BCZO with HPEC results in a platform that outperforms BC-ZnO alone, both in terms of physicochemical and biological functionality. This is further evidenced by the effective immobilization of Cc and Pp extracts within the BCZO/HPEC matrix (Scheme 2). The use of these natural bioactives introduces multifunctionality: anti-inflammatory action from Cc and antimicrobial/antioxidant effects from Pp, making the system promising for wound healing, biomedical coatings, or active food packaging.

To confirm the composite’s structure and integrity, a combination of SEM, EDS, XRD, and FTIR analyses was employed. These techniques not only validate the successful incorporation of ZnO into the cellulose framework but also provide insights into elemental distribution, crystallinity, and chemical interactions between the BC, ZnO, HPEC, and bioactive compounds.

#### Surface facial interface

**Fig. 4:**
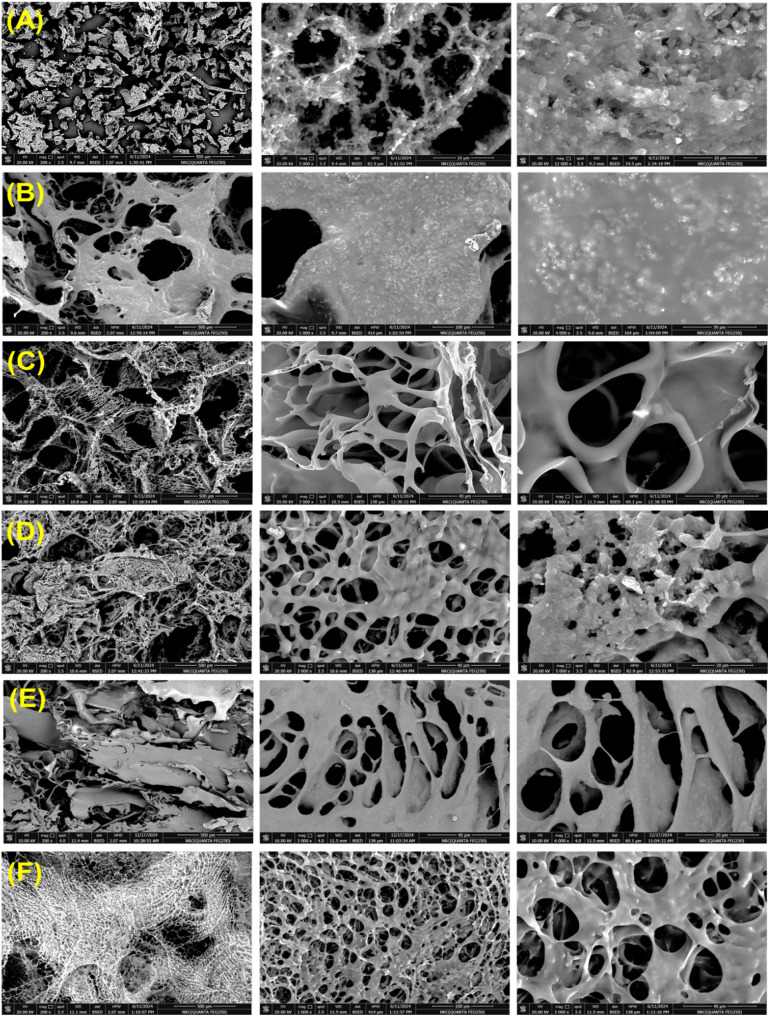
SEM micrographs of neat BCZO and functionalized BCZO-based composites at various magnifications: (**A**) neat BCZO at 200×, 5000×, and 12,000×, (**B**) BCZO/HPEC composite at 200×, 1000×, and 4000×, (**C**) Cc@BCZO/HPEC at 200×, 3000×, and 6000×, (**D**) Pp@BCZO/HPEC at 200×, 3000×, and 5000×, (**E**) Cc/Pp50@BCZO/HPEC at 200×, 3000×, and 6000×, and (**F**) Cc/Pp100@BCZO/HPEC at 200×, 1000×, and 3000×, respectively

SEM images of the dried and purified BCZO, along with its hybrid nanocomposite hydrogel, are presented in Fig. 4C–E. During the stirring phase, the suspension was visually monitored to ensure uniform opacity, the absence of agglomerates, and a consistent gel-like texture. The absence of phase separation or sedimentation upon brief static resting provided preliminary evidence of homogenization. To further confirm structural uniformity, SEM analysis of the freeze-dried samples revealed a well-dispersed distribution of BCZO nanoparticles within the Cc/Pp/HPEC matrix. The micrographs exhibited uniform spatial distribution with minimal clustering, verifying the effective incorporation of the BC-derived bionanoplatform components. The SEM micrographs (Fig. 4) offer comprehensive insight into the structural evolution of the fabricated nanocomposite scaffolds. Across all samples, the freeze-dried materials exhibit a characteristic interconnected nonwoven porous network, a direct consequence of the sublimation-driven solvent removal during lyophilization. This process leaves behind voids formerly occupied by ice crystals, which define the scaffold’s pore architecture, a phenomenon well-documented in porous material fabrication [60]. Importantly, this high porosity is preserved regardless of the inclusion of various functional additives such as HPEC, Cc, or Pp. This consistency suggests that the scaffold fabrication strategy maintains structural integrity even with added complexity, a promising trait for applications requiring controlled release, fluid permeability, or cellular infiltration.

At lower magnification, the BCZO aggregates are evident post-freeze-drying (Fig. 4A). Closer inspection reveals ZnO nanoparticles embedded and dispersed along the BC nanofiber network, forming a hybrid structure where the inorganic phase (ZnO) is well-integrated with the organic cellulose matrix. This indicates successful synthesis and suggests potential for synergistic mechanical and functional enhancements, such as antimicrobial or photocatalytic activity.

The BCZO/HPEC cross-section (Fig. 4B) displays a more uniform and coarse distribution of the nanocomposite across the surface, highlighting the role of HPEC as a stabilizing and dispersing agent. The even dispersion implies improved structural cohesion and possibly enhanced mechanical properties and biocompatibility due to HPEC’s known binding and emulsifying characteristics. Cross-sectional views of the functionalized composites, Cc@BCZO/HPEC, Pp@BCZO/HPEC, Cc/Pp50@BCZO/HPEC, and Cc/Pp100@BCZO/HPEC (Figs. 4C–F) reveal circular and ovaloid pores within a highly connected matrix. These pore morphologies, absent in pristine BC and BCZO samples, are indicative of the influence of the incorporated bioactives (Cc and Pp) on the scaffold microstructure. Such geometrical variations are valuable for tailoring fluid diffusion, drug release, or cell migration characteristics in biomedical applications.

At higher magnification, surface texture differences become apparent. The Pp@BCZO/HPEC scaffold shows a coarse, peeled surface, possibly due to the intrinsic waxy or resinous nature of Pp. In contrast, the Cc@BCZO/HPEC scaffold presents a smoother surface, reflecting Cc’s finer molecular dispersion. These differences persist in the mixed formulations (Cc/Pp50 and Cc/Pp100), suggesting that component ratio affects not just surface characteristics but also network fiber thickness and pore size.

Notably, thinner fiber networks and more refined pore distribution are observed in the Cc/Pp50@BCZO/HPEC and Cc/Pp100@BCZO/HPEC scaffolds. This indicates that dual loading may influence drying kinetics or intermolecular interactions during freeze-drying, resulting in a more compact matrix.

#### Elemental analysis

**Fig. 5:**
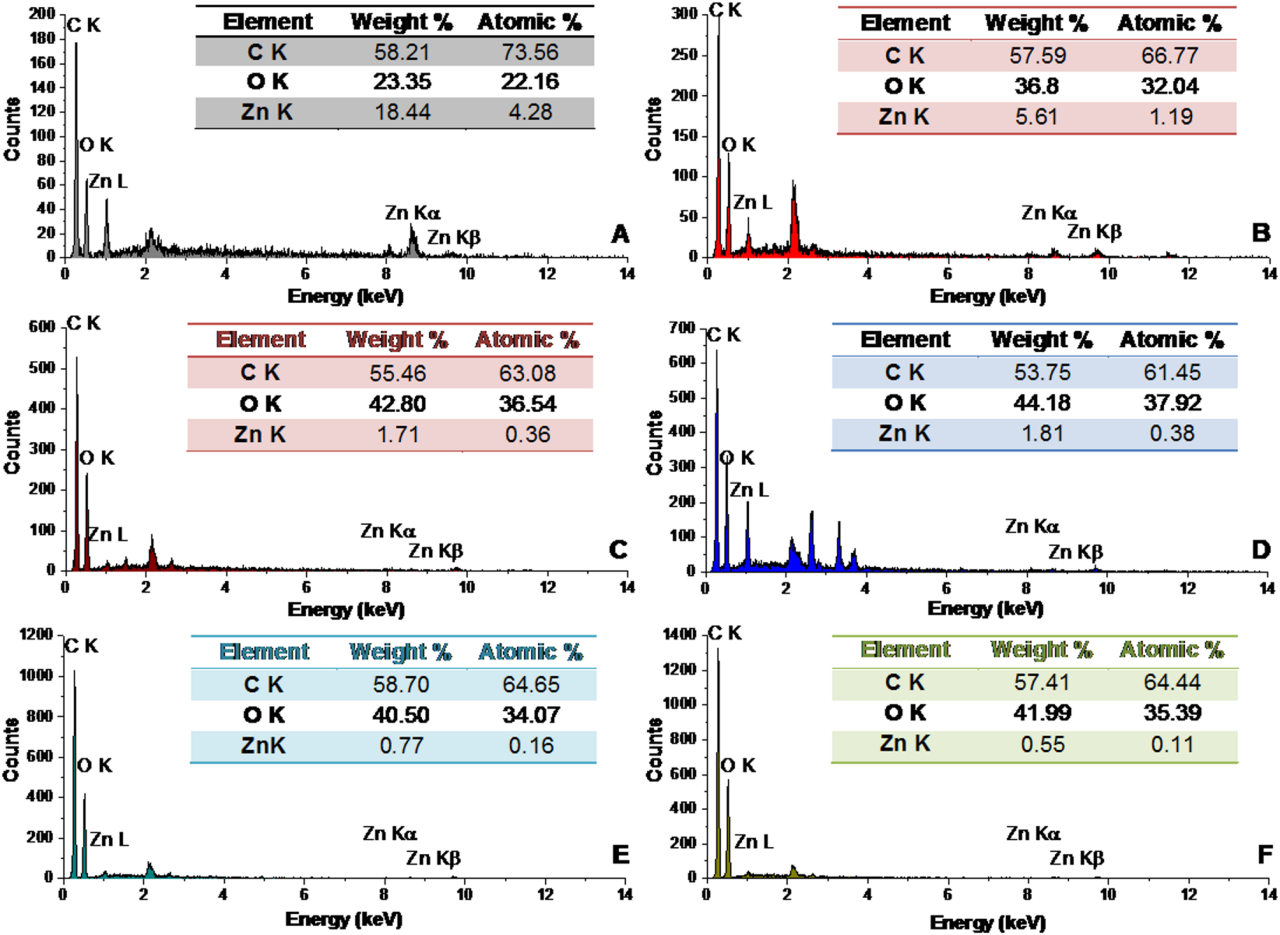
EDS of (**A**) BCZO, (**B**) BCZO/HPEC, (**C**) Cc@BCZO/HPEC, (**D**) Pp@BCZO/HPEC, (**E**) Cc/Pp50@BCZO/HPEC, and (**F**) Cc/Pp100@BCZO/HPEC Bionanoplatforms

Figure 5 presents the energy-dispersive X-ray spectroscopy (EDS) results for both the neat BCZO nanocomposite and its various hybrid nanoscaffold formulations. The EDS spectra confirm the presence of key elements, carbon (C Kα at ≈ 0.277 keV), oxygen (O Kα at ≈ 0.525 keV), and zinc (Zn Kα at ≈ 8.630 keV), with characteristic energy values matching those expected from standard X-ray emissions under electron beam excitation.

In the spectrum for neat BCZO, distinct peaks corresponding to C, O, and Zn were clearly observed, confirming the successful incorporation of ZnO nanoparticles into the BC matrix. Notably, the zinc content was relatively high at 18.44 wt%, indicating significant nanoparticle loading during synthesis.

In contrast, elemental analysis of the Cc/Pp100@BCZO/HPEC hybrid scaffold revealed a dramatic reduction in Zn content to 0.54 wt%, alongside an increased representation of carbon and oxygen. This decline is attributed to the dilution effect caused by the incorporation of HPEC, Cc, and Pp, all of which are organic, Zn-free compounds composed primarily of C, H, and O.

The sample BCZO/HPEC, which integrates hydroxypropyl ethyl cellulose (HPEC), showed a Zn content of 5.61 wt% and a corresponding increase in oxygen content to 36.8%, consistent with the molecular composition of HPEC [(C₂H₆O₂)ₙ]. This further confirms the impact of matrix composition on the relative elemental abundance observed via EDS.

Cc, a polyphenol with the formula C₂₁H₂₀O₆, lacks any metal elements and therefore does not introduce new elemental peaks in the EDS spectrum. Similarly, Pp, mainly composed of phenolic acids, flavonoids, and terpenes [61] contributes only to the C and O signals, reinforcing the trend of declining zinc signal intensity in scaffolds containing higher proportions of these bioactives.

Thus, the EDS data clearly illustrate that the progressive addition of HPEC, Cc, and Pp to the BCZO matrix dilutes the relative Zn concentration, without introducing foreign elemental peaks. These results not only confirm the successful compositional modification of the scaffolds but also highlight the chemical compatibility and integration of organic and inorganic components within the composite framework.

#### FTIR analysis

**Fig. 6:**
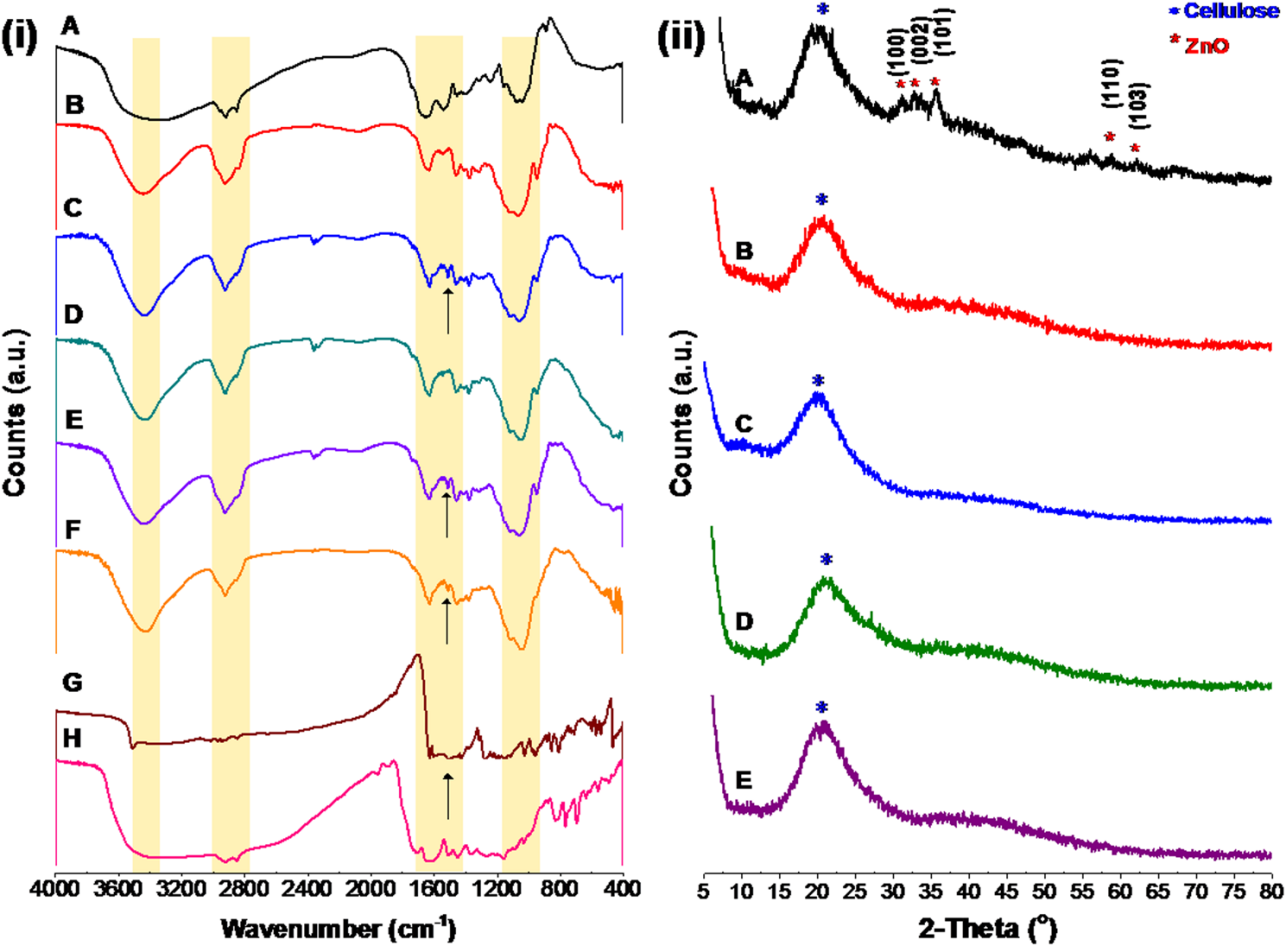
(i) FTIR of (**A**) BCZO, (**B**) BCZO/HPEC, (**C**) Cc@BCZO/HPEC, (**D**) Pp@BCZO/HPEC, (**E**) Cc/Pp50@BCZO/HPEC, and (**F**) Cc/Pp100@BCZO/HPEC (**G**) neat Cc, and (**H**) ethanolic Pp extract. (ii) XRD pattern of FTIR of (**A**) BCZO, (**B**) BCZO/HPEC, (**C**) Cc@BCZO/HPEC, (**D**) Pp@BCZO/HPEC, (**E**) Cc/Pp50@BCZO/HPEC, and (**F**) Cc/Pp100@BCZO/HPEC Bionanoplatforms

Figure 6 illustrates the FTIR spectra of BCZO, and BCZO/HPEC, composite with varying Cc and Pp weight percentages. The FTIR spectrum of BCZO validated the existence of fingerprint bands from the pristine BC (see Fig. 2B). The stretching vibrations of O–H and C–H exist at 3345 cm^− 1^, 2922 cm^− 1^, and 2850 cm^− 1^, respectively. A prominent band at 1652 cm^− 1^ signifies the bending vibrations of O–H bonds, likely associated with absorbed moisture in the sample. Two further bands at 1410 cm^− 1^ and 1313 cm^− 1^ exhibited CH’s symmetric deformation and bending vibrations, respectively. The band at 1038 cm^− 1^ is attributed to the stretching C–O–C bond, while the subdued peak at 530 cm^− 1^ is attributed to the Zn–O bond (Fig. 6i (A)) [37].

In Fig. 6B displays the FTIR spectrum of BCZO/HPEC composite. The absorption band of the composite at 3447 cm^− 1^ is attributed to the hydroxyl groups from the BC and HPEC pyranose unit. The absorption bands at 2925 cm^− 1^ and 2835 cm^− 1^ correspond to CH_2_ and CH stretching vibrations, respectively. The absorption bands at 1547 cm^− 1^ and 1066 cm^− 1^ are attributed to the C-H bending and C-O-C stretching vibrations, respectively. The FTIR spectra of BCZO/HPEC composites loaded with scaffolds with Cc and Pp (Fig. 6C-F) reveal all the distinctive fingerprint bands of the cellulose structure from both BC and HPEC. The sole distinction is the appearance of a new peak at 1511 cm^− 1^ (shown by the blue arrow) in the BCZO/HPEC scaffolds incorporating Cc in its composition. This peak is attributed to the absorption of C = C in the stretching vibration mode within the Cc structure [62]. All data suggest that the HPEC, Cc, and Pp were sucessfuly imopilized into BCZO/HPEC which interact together via the intermolecular hydrogen bondings.

#### XRD analysis

XRD serves as a vital analytical tool for elucidating the structural organization and phase distribution within bio-nanocomposites. It provides essential insights into crystallinity, phase composition, and possible intercalation or exfoliation of nanoparticles within the polymeric matrix. This is particularly important for understanding how embedded nanomaterials like ZnO interact with and influence the structure of biopolymers such as BC.

The XRD pattern of BCZO (Fig. 6ii-A) reveals the persistence of BC’s characteristic crystalline planes at 2θ = 19.25° (110) and 2θ = 21.54° (020), which are associated with the cellulose II (CII) allomorph. These peaks remain largely unaltered compared to native BC (Fig. 1C), aside from slight shifts to higher 2θ values (19.88° and 21.68°), likely due to internal stresses or minor structural rearrangements following ZnO incorporation. The retention of BC’s crystallinity suggests that ZnO deposition did not significantly disrupt the native BC crystal lattice.

Notably, the presence of additional diffraction peaks at 31.02°, 32.97°, 35.56°, 58.79°, and 62.08° confirms the formation of crystalline ZnO nanoparticles. These peaks correspond to the (100), (002), (101), (110), and (103) planes of the hexagonal wurtzite structure of ZnO, as referenced by JCPDS card No. 36-1451 [63]. Minor deviations in peak positions may be attributed to variations in sample preparation, particle size, or instrumental parameters.

However, upon the integration of BCZO with HPEC, Cc, and Pp to form various hybrid nanocomposites (Fig. 6ii, B–E), the ZnO-related peaks disappear, while the primary BC peaks are retained. This observation may result from several factors:

i. Low ZnO concentration, as supported by EDS analysis (Fig. 5), causes the Zn content in some composites to drop below 2 wt%, which may fall below the XRD detection threshold, especially when embedded in a matrix dominated by amorphous or weakly crystalline polymers.
ii. Peak masking or overlap, the strong and broad diffraction signals from the polymer matrix (BC, HPEC, or bioactive) may obscure weaker ZnO peaks, particularly if the ZnO particles are well-dispersed or confined at the nanoscale.
iii. Particle size and dispersion, if ZnO particles are tiny (< 10 nm) or highly dispersed, they may yield broad, weak diffraction signals that are indistinguishable from background noise or polymeric peaks.

These findings imply that while ZnO nanoparticles are present and structurally integrated within the BC-based matrix, as confirmed by TEM and EDS, their crystalline signature is not prominently expressed in the hybrid scaffold’s XRD pattern. This supports the hypothesis that during the composite formation, strong interactions and structural embedding of ZnO within the BCZO/HPEC/Cc/Pp matrix may occur, potentially modifying the nanoparticles’ crystalline expression.

### Biological evaluation of the fabricated scaffolds

To assess the multifunctional biological performance of the developed bionanoplatforms, various formulations were prepared by incorporating ZnO@BC, HPEC, and bioactive agents such as Pp and Cc. Table 1 summarizes the composition of these formulations, detailing the quantities of each component along with the calculated drug loading (%) and encapsulation efficiency (%). Drug loading was determined using the weight difference method based on the Judefeind and de Villiers equation (Eq. 1). Encapsulation efficiency was assumed to be 100%, attributed to the lyophilization process, which minimizes material loss. The resulting platforms, containing either single or combined drugs (Cc and/or Pp), exhibited drug loading values ranging from 10 to 20%, directly reflecting the amount of drug incorporated into the BCZO/HPEC matrix. These well-characterized nanocomposites were subsequently evaluated for antimicrobial activity against representative bacterial strains, antioxidant capacity, cytotoxicity, and in vitro wound healing performance using scratch assays.

#### Antimicrobial activity

**Fig. 7:**
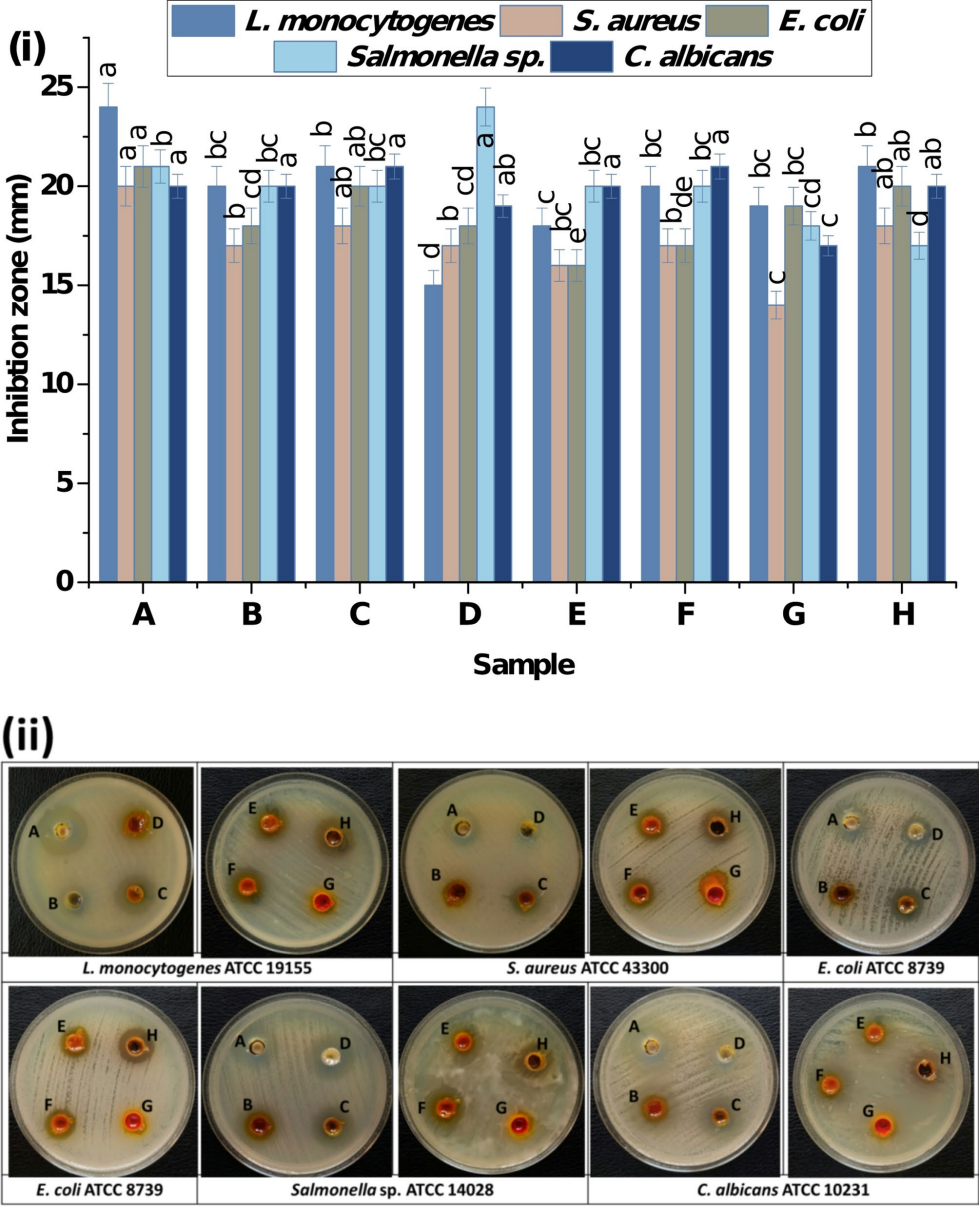
Antimicrobial activities of (**A**) BCZO, (**B**) BCZO/HPEC, (**C**) Cc@BCZO/HPEC, (**D**) Pp@BCZO/HPEC, (**E**) Cc/Pp50@BCZO/HPEC, (**F**) Cc/Pp100@BCZO/HPEC, (**G**) Cc, and (**H**) Pp, against *L. monocytogenes* ATCC 19155, *S. aureus* ATCC 43300, *E. coli* ATCC 8739, *Salmonella sp*. ATCC 14028 and *C. albicans* ATCC 10231. *a, b values in the above column with the same letter do not differ significantly according to Duncan’s test at the 5% level. Bar indicated to ± standard deviation

The antimicrobial potency of the as-prepared scaffold was studied using agar diffusion method against *L. monocytogenes* ATCC 19155, *Staphylococcus aureus* ATCC 43300, Gram-negative bacteria *E. coli* ATCC 8739, *Salmonella* sp. ATCC 14028, and unicellular fungi *Candida albicans* ATCC 10231. Figure 7 displays the antimicrobial activity against the tested microorganisms, and the results are summarized as follows:

i. BCZO Bionanoplatforms: The BCZO bionanoplatforms displayed significant potency in killing and inhibiting a variety of multidrug-resistant microorganisms. This strong antimicrobial effect is principally due to the decoration of BC with ZnO nanoparticles, which possess broad-spectrum antimicrobial properties. When BCZO was immobilized into hydroxypropyl ethyl cellulose (HPEC) at a 1:9 (wt/wt) ratio, forming the BCZO/HPEC platform, it maintained comparable activity against *Staph. aureus* (with inhibition zones of 17 mm versus 20 mm for neat BCZO) and even enhanced activity against *Listeria monocytogenes* (24 mm versus 20 mm). Also, its effectiveness against *E. coli* and Salmonella sp. was moderately reduced (18 and 20 mm compared to 21 and 21 mm, respectively), likely due to the decreased ZnO content in the composite.
ii. Incorporation of Pp or Cc: When either Pp or Cc was immobilized onto the BCZO/HPEC platform, the antimicrobial potency improved further. Both the Cc-containing and the Pp-containing platforms exhibited almost identical inhibition zones across the tested strains. For instance, the Cc platform achieved zones of 21, 18, 20, 20, and 21 mm, while the Pp platform obtained 15, 17, 18, 24, and 19 mm. This similarity suggests an additive effect, whereby both Cc and Pp enhance the antimicrobial activity provided by the ZnO nanoparticles.
iii. Combined Immobilization of Pp and Cc: Interestingly, when both Pp extract and Cc were co-immobilized onto the BCZO/HPEC platform, at concentrations of 50 and 100 mg/g, respectively, the resulting platforms (Cc/Pp@BCZO/HPEC) exhibited nearly identical microbicidal activity against all the tested strains. The measured inhibition zones were 18, 16, 16, 20, and 20 mm versus 20, 17, 17, 20, and 21 mm for *L. monocytogenes*, *S. aureus*, *E. coli*, *Salmonella* sp., and *C. albicans*, respectively.

The mechanism of antimicrobial activity for the fabricated bionanoscafolds can be explained based on these facts. ZnO nanoparticles exert their bactericidal effect primarily by releasing Zn²⁺ ions, which compromise the integrity of bacterial cell membranes and disrupt critical metabolic pathways [64]. As a class II–V semiconductor transition metal oxide with a wide band gap of 3.3 eV, ZnO facilitates the generation of reactive oxygen species (ROS). This occurs by oxidizing water molecules or hydroxide anions, thereby initiating a redox chain reaction that produces a variety of ROS, namely hydroxyl, hydroperoxide, and superoxide radical anions (O₂•⁻). The resultant oxidative stress in bacterial cells inhibits protein synthesis and DNA replication, ultimately leading to cell death [65].

Pp extract is abundant in diverse natural bioactive compounds, including prenylated molecules, such as artepillin C (3,5-diprenyl-pcoumaric acid), 3prenylcinnamic acid allyl ester, 2dimethyl-8prenylchromene, and other prenyl derivatives, as well as flavonoids like pinocembrin and apigenin. These components confer potent antibacterial activity against methicillin-resistant *Staphylococcus aureus* (MRSA) by disrupting the bacterial membrane potential and inhibiting ATPase, which in turn impairs bacterial motility, cell division, and biofilm formation [66]. Similarly, Cc is a natural extract derived from *Curcuma longa*; it is well known for its antimicrobial, anti-inflammatory, antioxidant, and anticancer properties. Its antimicrobial actions arise from membrane disruption, bacterial DNA damage, and induced membrane leakage, leading to the generation of reactive oxygen species such as singlet oxygen and hydroxyl radicals [67]. Additionally, the polyphenolic structure of Cc is fundamental to its antioxidant activity, further enhancing its therapeutic potential [30].

#### Antioxidant activity

**Fig. 8:**
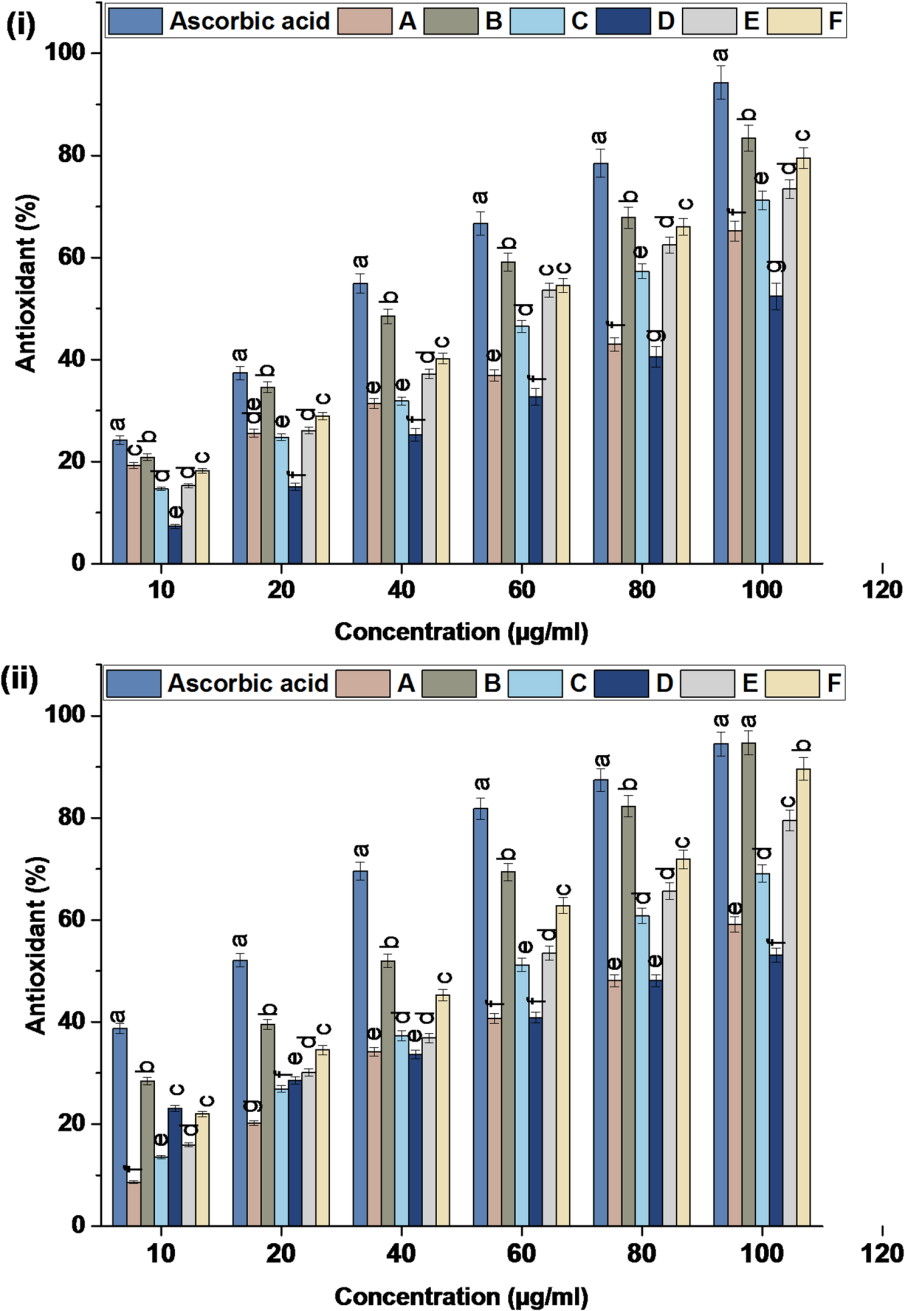
Antioxidant activity of (**A**) BCZO, (**B**) BCZO/HPEC, (**C**) Cc@BCZO/HPEC, (**D**) Pp@BCZO/HPEC, (**E**) Cc/Pp50@BCZO/HPEC, and (**F**) Cc/Pp100@BCZO/HPEC Bionanoplatforms using (i) DPPH assay, (ii) ABTS assay. * a, b values in the above column with the same letter do not differ significantly according to Duncan’s test at the 5% level. Bar indicated to ± standard deviation

In this study, the antioxidant activity of various bacterial cellulose-derived platforms was evaluated using DPPH and ABTS assays at concentrations ranging from 10 to 100 µg/mL (Fig. 8; Table 2). Notably, platforms containing Cc exhibited significantly higher antioxidant activity compared to those formulated with only Pp extract or ZnO nanoparticles. IC₅₀ values for Cc@BCZO/HPEC recorded 54.86 ± 0.29 and 60.39 ± 0.33 where as IC₅₀ values for Pp@BCZO/HPEC gave 97.15 ± 0.56 and 103.50 ± 0.59 using DPPH and ABTS assays respectively. At concentrations between 40 and 100 µg/mL, the Cc/BCZO/HPEC platform demonstrated free-radical scavenging capabilities greater than 50%, a performance comparable to that of ascorbic acid. Additionally, incorporating both Cc and Pp into BCZO resulted in Cc/Pp50/BCZO/HPEC and Cc/Pp100/BCZO/HPEC formulations that yielded high antioxidant activity, with IC₅₀ values of 47.48 ± 0.25 and 51.53 ± 0.26 µg/mL for the former, and 35.65 ± 0.21 and 45.65 ± 028 µg/mL for the latter using DPPH and ABTS assays respectively.

Cc, the principal bioactive compound found in turmeric (*Curcuma longa*), is widely recognized for its potent antioxidant properties. Its chemical structure, characterized by conjugated double bonds and multiple phenolic hydroxyl groups, enables Cc to effectively donate electrons and neutralize free radicals, such as reactive oxygen species (ROS). This free-radical scavenging activity not only protects cellular components from oxidative damage but also modulates the activity of antioxidant enzymes [68].

Pp exhibits significant therapeutic potential for injury management due to its complex composition of approximately 420 bioactive constituents [69], predominantly phenolic compounds, flavonoids, and phenolic acids. These compounds confer a wide range of biological activities, including antimicrobial [70], antioxidant [71], and anti-inflammatory effects [72].

The incorporation of Pp extract into various polymeric matrices such as polycaprolactone (PCL), cellulose acetate, pectin, and chitosan has been shown to enhance the antioxidant properties of these materials. This enhancement, evaluated via DPPH radical scavenging, correlated positively with increasing extract concentration. The antioxidant efficacy of Pp has been well-documented in prior studies [73], primarily attributed to its phenolic constituents.

**Table 2:** IC_50_ for cytotoxicity and IC_50_ for antioxidant activity of BCZO bionanoplatforms

Sample	IC_50_ for cytotoxicity	IC_50_ for antioxidant activity	
	µg/mL	DPPH assay	ABTS assay
		µg/mL	µg/mL
BCZO	354.29 ± 2.4	78.64 ± 0.42	84.38 ± 0.47
BCZO/HPEC	337.73 ± 0.49	27.46 ± 0.16	37.39 ± 0.24
Cc@BCZO/HPEC	354.46 ± 2.87	54.86 ± 0.29	60.39 ± 0.33
Pp@BCZO/HPEC	350.39 ± 1.87	97.15 ± 0.56	103.50 ± 0.59
Cc/Pp50@BCZO/HPEC	333.45 ± 0.83	47.48 ± 0.25	51.53 ± 0.26
Cc/Pp100@BCZO/HPEC	162.28 ± 0.39	35.65 ± 0.21	45.65 ± 0.28

#### Cytotoxicity assessment of the fabricated scaffolds

**Fig. 9:**
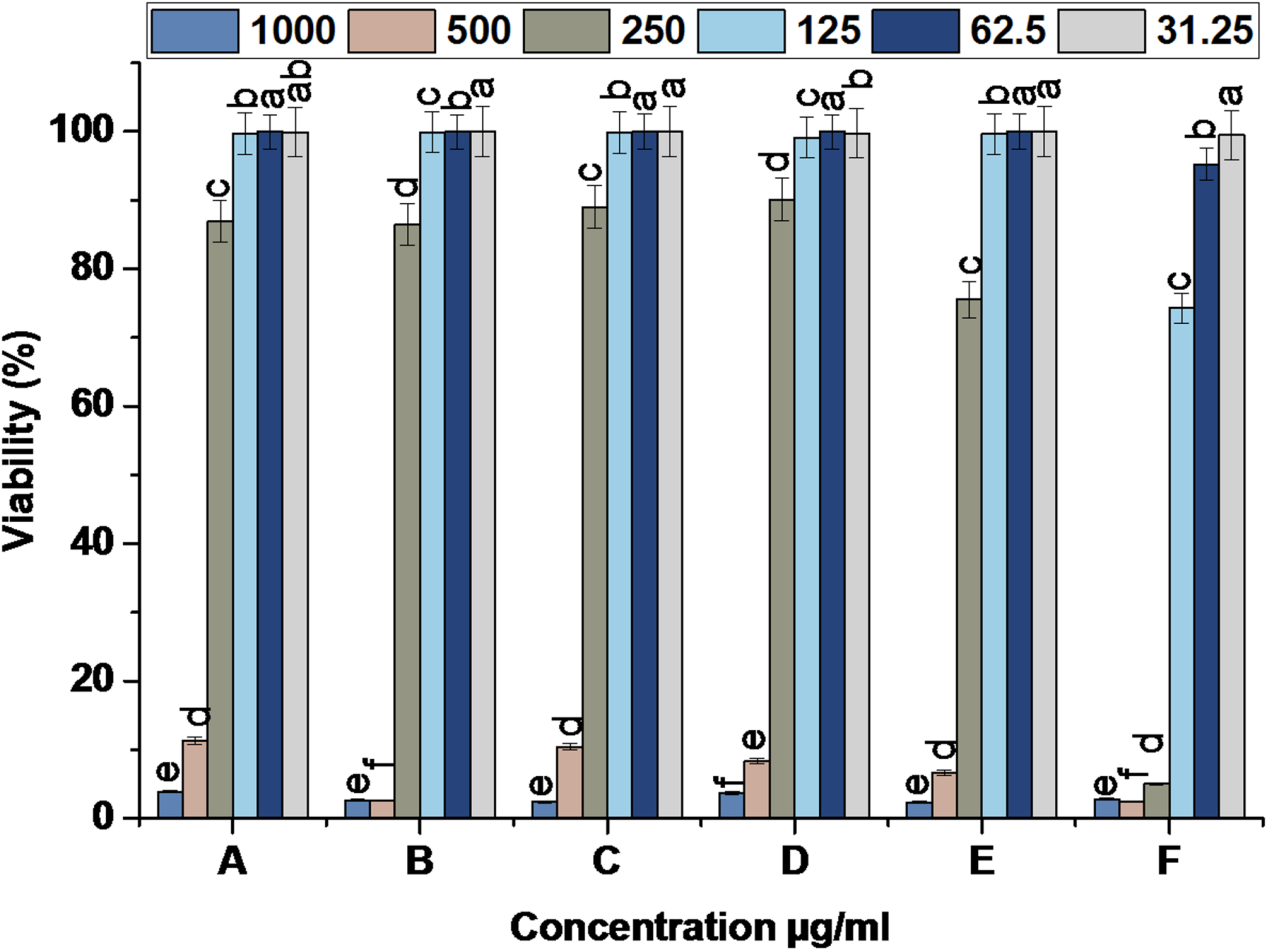
Cytotoxicity of (**A**) BCZO, (**B**) BCZO/HPEC, (**C**) Cc@BCZO/HPEC, (**D**) Pp@BCZO/HPEC, (**E**) Cc/Pp50@BCZO/HPEC, and (**F**) Cc/Pp100@BCZO/HPEC Bionanoplatforms, against HFB-4 cell line. * a, b values in the above column with the same letter do not differ significantly according to Duncan’s test at the 5% level. Bar indicated to ± standard deviation

The scaffold’s cytocompatibility towards HFB-4 normal skin cell line was assessed, as shown in Fig. 9 and Supplementary Fig. 1. All composites with BC, except Cc/Pp100@BCZO/HPEC exhibited negligible toxicity against normal fibroblast skin cells at composite concentrations less than 250 µg/mL, demonstrating excellent biocompatibility with > 99% cell viability [74]. All scaffolds have severe toxicity at a concentration exceeding 500–1000 µg/mL. IC50 for BCZO, BCZO/HPEC, Cc@BCZO/HPEC, Pp@BCZO/HPEC recorded 354.29 ± 2.4, 337.73 ± 0.49, 354.46 ± 2.87, and 350.39 ± 1.87 µg/mL, respectively. Meanwhile, the two scaffolds Cc/Pp50@BCZO/HPEC and Cc/Pp100@BCZO/HPEC showed a mild toxicity at 250 µg/mL with IC50 333.45 ± 0.83 and 162.28 ± 0.39 µg/mL, respectively. The biocompatibility, antioxidant activity, and broad-spectrum antimicrobial activity strongly endorse the possible utility of different scaffolds in biomedical field applications, including implants, wound dressing, and scaffolds for tissue regeneration [28, 75] .

#### Application of selected scaffolds in in vitro wound healing assessment

Given its excellent antimicrobial, antioxidant, and cytocompatibility properties, the Cc/Pp50@BCZO/HPEC scaffold was chosen to evaluate its potential in mediating skin wound healing. This composite scaffold incorporates ZnO nanoparticles and a Pp extract that exhibit significant antimicrobial activity, while Cc contributes robust antioxidant effects and suppresses the inflammatory response associated with skin injury.

**Fig. 10:**
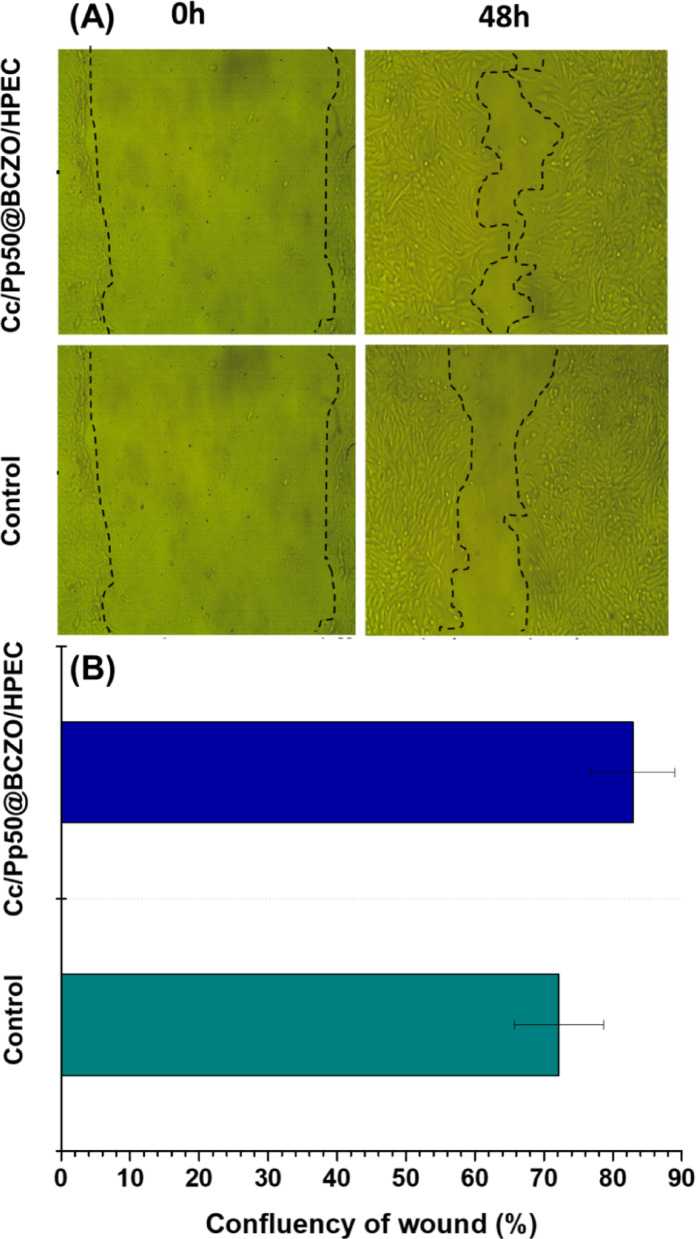
**A** Fluorescence images from the wound healing assay of Cc/Pp50@BCZO/HPEC at 0 h and 48 h using a ZOE Fluorescent Cell Imager (BIO-RAD, USA). **B** Wound confluency (%) was quantified via ImageJ software, with results expressed as mean ± SD from triplicate experiments

Cc/Pp50@BCZO/HPEC was evaluated using an in vitro scratch assay on normal human skin fibroblast (HFB-4) cells. Subsequent imaging at various time intervals revealed that the active compounds in Cc/Pp50@BCZO/HPEC effectively stimulated the repair process, as evidenced by the progressive merging of the cell fronts to close the gap (Fig. 10A).

Detailed examination of the scratch indicated an accelerated wound healing response mediated by Cc/Pp50@BCZO/HPEC by reducing the gap from both edges of the wound. The scratch examination after treatment with Cc/Pp50@BCZO/HPEC demonstrated high impact on the scratch closure process, reducing the gap from both sides with wound confluences of 82.91% as compared with control (72.19%) (Fig. 10B). These observations are in line with previous research showing that bacterial cellulose nanocomposites integrated with different nanoparticles possess inherent wound-healing properties [68]. In particular, zinc oxide nanoparticles are known not only for their broad-spectrum antimicrobial activity but also for their ability to promote cell migration, reepithelialization, and angiogenesis during the healing process [76].

Additionally, bacterial cellulose itself supports wound healing due to its unique attributes, including a uniform nanofiber network, high crystallinity, and excellent water absorption and retention capabilities [77]. Together, these properties make the Cc/Pp50@BCZO/HPEC platform a promising material for facilitating effective tissue repair and skin regeneration.

## Conclusion

Rotten fruits were successfully utilized as a sustainable biomass source for the production of bacterial cellulose (BC) using a novel strain identified via 16 S rRNA sequencing as *Limosilactobacillus fermentum* 6BC. The structure of the resulting BC was confirmed by TEM, FTIR, and XRD analyses. ZnO nanoparticles were synthesized via a sono-coprecipitation method and uniformly deposited onto the BC matrix, forming a ZnO-decorated BC (BCZO) nanocomposite. This nanocomposite was subsequently integrated into a hydroxypropyl ethyl cellulose (HPEC) matrix to form a multifunctional platform.

To enhance the biological properties, curcumin (Cc) and proplise (Pp) were successfully immobilized onto the composite, yielding bioactive scaffolds with antibacterial, antioxidant, and wound healing capabilities while maintaining excellent biocompatibility. The BCZO/HPEC system demonstrated a highly functional, structurally robust, and tunable bionanocomposite configuration. The strategic integration of nanostructured ZnO and phytochemicals within the biocompatible cellulose-based matrix contributed to its multifunctionality, bioactivity, and processability.

SEM analysis confirmed the successful fabrication of highly porous and morphologically diverse structures, with tunable microarchitecture influenced by the composition and ratio of incorporated agents. These structural features are expected to impact key performance metrics, including drug loading and release behavior and biological interactions. XRD patterns revealed the crystalline nature of ZnO in neat BCZO, while its peaks were attenuated or absent in hybrid formulations due to low content, nanoscale dispersion, and possible overlap with the polymer matrix, indicating effective incorporation and potential structural reorganization.

All fabricated scaffolds exhibited broad-spectrum antimicrobial and antioxidant activities, arising from the synergistic effects of ZnO NPs, Cc, and Pp. Notably, the antioxidant capacity of Cc-loaded scaffolds was comparable to that of ascorbic acid. The Cc/Pp50@BCZO/HPEC formulation showed promising wound healing efficacy, significantly enhancing the closure of scratch wounds in vitro.

Overall, the BCZO/HPEC-based scaffolds demonstrated excellent multifunctionality, including biocompatibility, antimicrobial performance, antioxidant activity, and regenerative potential. These results validate their application in biomedical fields such as wound dressings, implants, and tissue engineering scaffolds. The platform offers versatility and customization potential, making it a promising candidate for next-generation biofunctional materials.

For future work, comprehensive in vivo studies should be conducted to evaluate wound closure dynamics, inflammatory responses, and tissue regeneration outcomes. Furthermore, exploring stimuli-responsive release systems (e.g., pH- or enzyme-triggered release) could enhance the precision of therapeutic delivery. Expanding the range of bioactive compounds, including antimicrobial peptides or growth factors, may further broaden the platform’s applicability. Finally, scaling up the production process and assessing industrial feasibility and regulatory compliance will be essential for translating this platform into practical biomedical and commercial applications.

## Supplementary Information

Additional file 1.

## Data Availability

No datasets were generated or analysed during the current study.
