# Supplementary material for: Bacterial cellulose doped with ZnO as a multifunctional bioactive platform for curcumin and propolis immobilization: synthesis, characterization, and wound healing potential
Source: Microb Cell Fact. 2025 Aug 25;24:196. doi: 10.1186/s12934-025-02826-6 (PMC12376435; doi:10.1186/s12934-025-02826-6)
Supplement: Supplementary file 1 — Additional file 1. [file 12934_2025_2826_MOESM1_ESM.docx]

**Bacterial Cellulose Doped with ZnO as a Multifunctional Bioactive Platform for Curcumin and Propolis Immobilization: Synthesis, Characterization, and Wound Healing Potential**

**Ghada E. Dawwam^a*^, Naglaa Salem El-Sayed^b^, Mona T. Al-Shemy^b^**

^a^Botany and Microbiology Department, Faculty of Science, Benha University, Benha 13518, Egypt

^b^Cellulose and Paper Department, National Research Centre, 33 El-Bohouth St. (Former El-Tahrir St.), Dokki, P.O. 126220, Giza, Egypt.

***Corresponding author:** ghada.ibrahem@fsc.bu.edu.eg. https://orcid.org/0000-0002-2911 658X

| 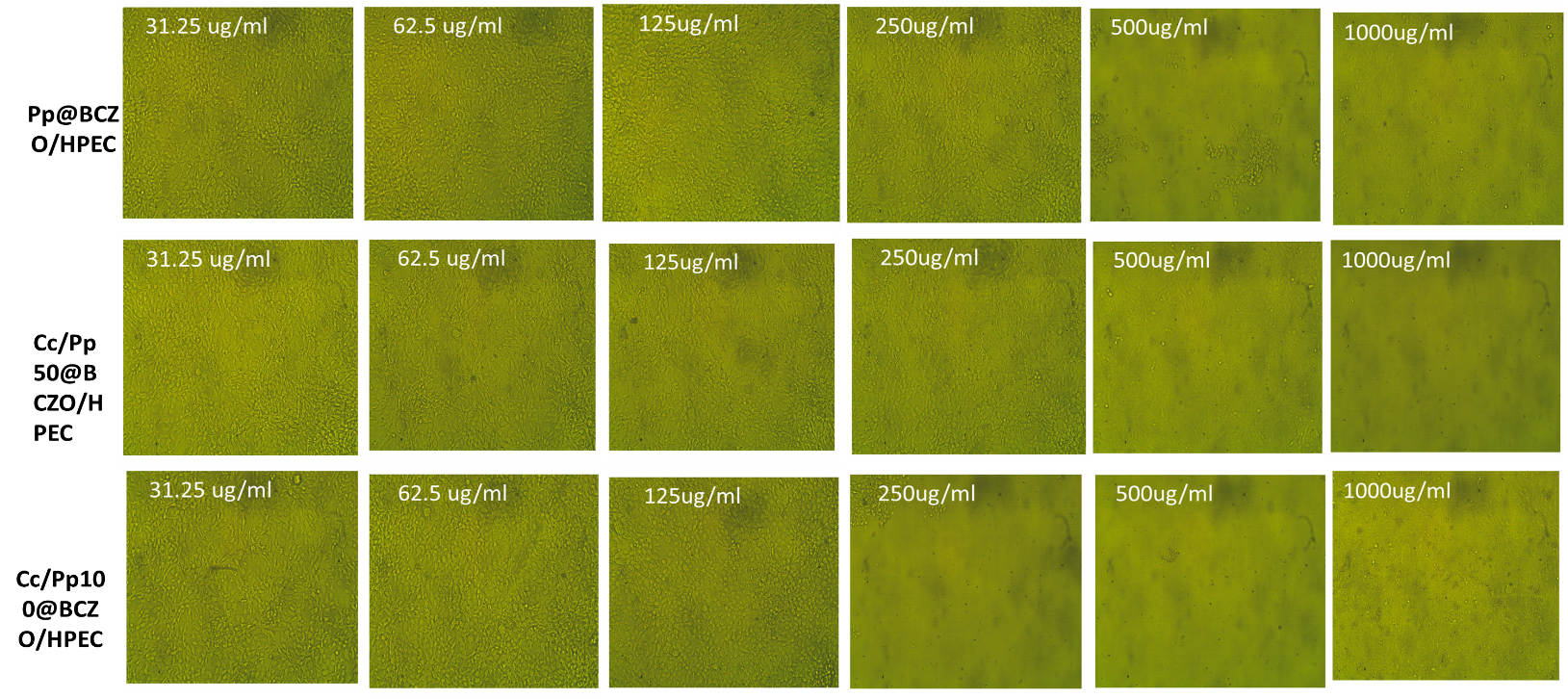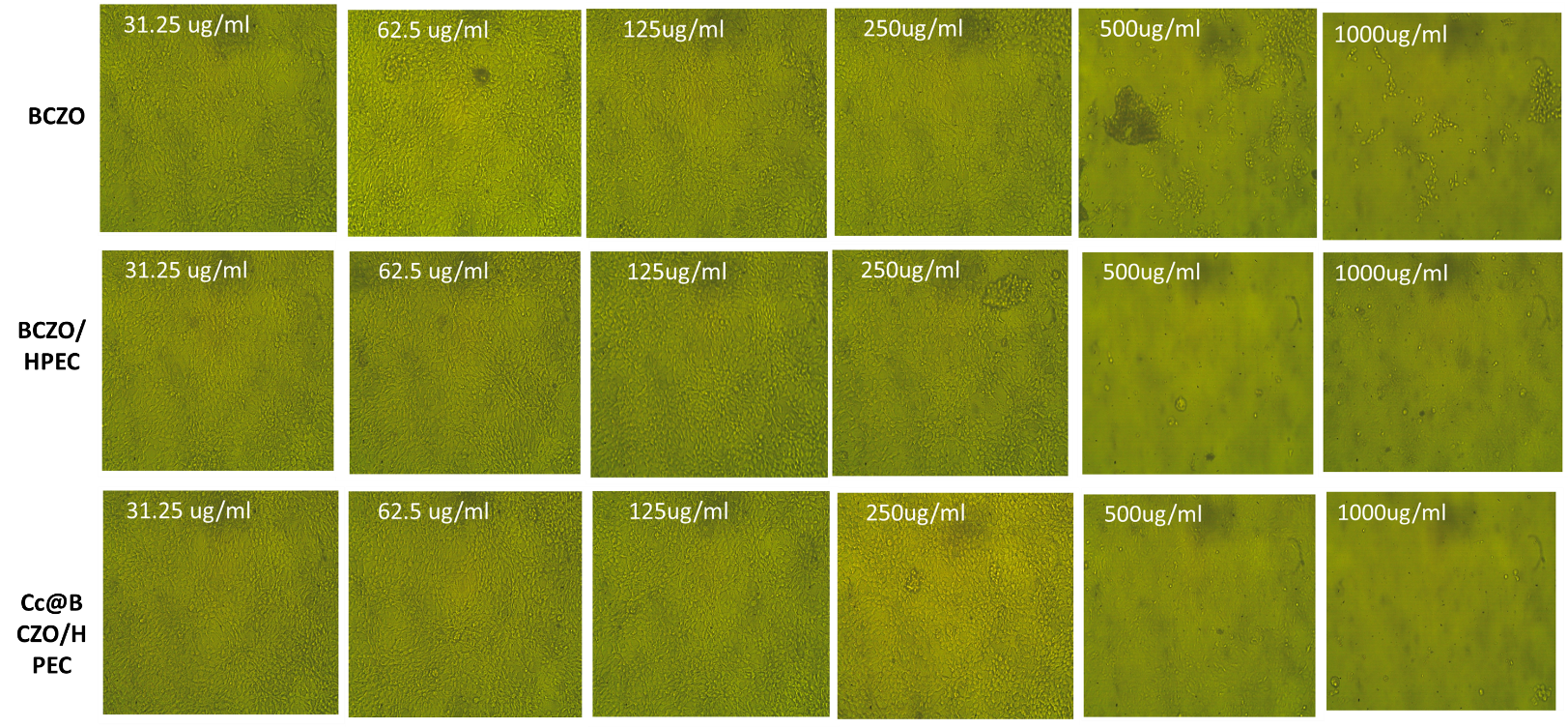  **Supplementary Figure 1:** Cytotoxicity of different Bionanoplatforms against HFB-4 cell line. |
| --- |
